# Effectiveness of eHealth Interventions Promoting Physical Activity in Children and Adolescents: Systematic Review and Meta-Analysis

**DOI:** 10.2196/41649

**Published:** 2024-02-21

**Authors:** Irene Sequí-Domínguez, Iván Cavero-Redondo, Celia Álvarez-Bueno, Jose Francisco López-Gil, Vicente Martínez-Vizcaíno, Carlos Pascual-Morena

**Affiliations:** 1 Health and Social Research Center, Universidad de Castilla-La Mancha Cuenca Spain; 2 Research Network on Chronicity, Primary Care and Health Promotion Cuenca Spain; 3 Facultad de Enfermería de Albacete, Universidad de Castilla-La Mancha Albacete Spain; 4 Facultad de Ciencias de la Salud, Universidad Autónoma de Chile Talca Chile; 5 Universidad Politécnica y Artística del Paraguay Asunción Paraguay; 6 One Health Research Group Universidad de las Americas Quito Ecuador

**Keywords:** eHealth technologies, physical activity, sedentary behaviors, children, mobile phone

## Abstract

**Background:**

eHealth interventions have been postulated as a feasible, acceptable, and possibly effective tool to promote physical activity (PA) among children and adolescents; however, a comprehensive quantitative analysis of the effects of eHealth interventions promoting PA is lacking.

**Objective:**

This study aims to conduct a systematic review and meta-analysis on experimental studies reporting the effects of eHealth interventions aimed at promoting PA on PA parameters and sedentary behavior parameters in children and adolescents.

**Methods:**

The CENTRAL, MEDLINE, Embase, and Web of Science databases were searched from inception to February 2022 for randomized controlled trials that analyzed the effects of eHealth interventions aimed at promoting PA on PA and sedentary parameters in children and adolescents. The Hartung-Knapp-Sidik-Jonkman random effects method was used to determine the mean differences (MDs) with their respective 95% CIs. The risk of bias was assessed using the Risk of Bias 2 (RoB2; Cochrane) tool and its extension for cluster randomized controlled trials. The certainty of evidence was evaluated using the Grading of Recommendations, Assessment, Development, and Evaluation (GRADE) tool.

**Results:**

A total of 20 trials reporting the effects of different eHealth interventions aimed at promoting PA were included. Results for each parameter were as follows: counts per minute (MD −16.11 counts, 95% CI −122.76 to 90.53; *k*=3; n=402; *I*^2^=69%; favoring control), steps per day (MD 593.46 steps, 95% CI −2102.27 to 3289.19; *k*=2; n=152; *I*^2^=0%; favoring intervention [FI]), moderate to vigorous PA (MD −1.99 min/d, 95% CI −8.95 to 4.96; *k*=14; n=2336; *I*^2^=86%; favoring control), light PA (MD 3.28 min/d, 95% CI −15.48 to 22.04; *k*=5; n=355; *I*^2^=67%; FI), screen time (MD −31.48 min/d, 95% CI −68.62 to 5.65; *k*=5; n=904; *I*^2^=0%; FI), and sedentary time (MD −33.12 min/d, 95% CI −57.27 to −8.97; *k*=8; n=819; *I*^2^=75%; FI). Our results should be interpreted cautiously because of important limitations such as the scarcity of evidence, overall risk of bias, and low to very low certainty of evidence.

**Conclusions:**

We did not find conclusive evidence regarding the impact of PA-targeted eHealth interventions on PA parameters, but the very low certainty of evidence suggests that eHealth interventions may reduce sedentary time in children and adolescents. Our results may have important scientific implications as they highlight that the rapid development of eHealth interventions to promote PA lacks robust supporting evidence.

**Trial Registration:**

PROSPERO CRD42020211020; https://www.crd.york.ac.uk/prospero/display_record.php?RecordID=211020

## Introduction

Physical inactivity is considered one of the most important modifiable risk factors for noncommunicable diseases, as 1.6 million deaths per year can be attributed to insufficient physical activity (PA) [1]. Moreover, physical inactivity is associated with an increased risk of cardiovascular disease, hypertension, type 2 diabetes, multiple types of cancer, dementia, depression, as well as cardiovascular disease–related mortality and all-cause mortality in adults [2,3]. As childhood PA-related behaviors are associated with cardiometabolic risk and tend to endure from childhood through adolescence into adulthood [4,5], the promotion of PA at early ages is not only a cardiometabolic prevention strategy but can also be considered an adult prevention intervention [6-8].

According to the World Health Organization guidelines, children and adolescents (aged 5 to 17 years) should engage in an average of 60 minutes of moderate to vigorous PA (MVPA) per day and limit their sedentary time. In addition, these guidelines recommend that vigorous PA and muscle- and bone-strengthening activities should each be incorporated at least 3 days per week [9,10]. However, approximately 70% of this age group do not meet these recommendations [11]. Consequently, the intervention strategies for PA promotion in children and adolescents remain an important public health topic. Understanding waking activities as a continuum, where sedentary behaviors are waking activities characterized by low energy expenditure and performed in a sitting or reclining posture [12], interventions aimed at increasing energy expenditure during waking time may be able to increase PA while diminishing sedentary time.

Despite evidence supporting the substantial health benefits of engaging in adequate levels of PA and decreasing sedentary behaviors during childhood [13-15], interventions promoting PA targeting children and adolescents have shown serious shortcomings, including a lack of scalability, high costs, or difficulties in sustaining or implementing them [16]. Therefore, finding feasible, scalable, and effective interventions to encourage children and adolescents to adopt active lifestyles is a public health priority [17].

eHealth is defined as a cost-effective and safe use of information and communication technologies in support of health and health-related domains, enabling better communication between practitioners and patient practitioners, better monitoring and data management, and acting as a vehicle to deliver health information and interventions for prevention and care [18]. eHealth has been postulated as a feasible, acceptable, and possibly effective tool for promoting PA among children and adolescents [19]. The potential of eHealth interventions to engage young population not only resides in the fact that they are *digital natives* but also on the characteristics and diverse possibilities of eHealth delivery; these include features such as allowance for tailored feedback, monitoring, direct interaction, report on goals and track of progress, or access to a community [19,20].

Previous systematic reviews and meta-analyses studying the effects of mobile health (mHealth) technologies [21-28], SMS text message interventions [29], and digital interventions [30] in children and adolescents on PA and sedentary behavior parameters are inconclusive. However, when the horizon is broadened to analyze eHealth interventions aimed at promoting PA as a whole, they are postulated as a successful approach to increase PA; however, there is a lack of quantitative synthesis to support such conclusions [19,20]. This makes it difficult to make a pronouncement on the pillars of solid evidence as to whether the eHealth interventions that promote PA can be more effective than traditional PA promotion interventions.

Furthermore, owing to the scarcity and heterogeneity of available data, previous evidence [19-30] supports the necessity for more comparable studies to accumulate meaningful evidence.

The most recent study [22] showed the most promising results by only analyzing smartphone-based interventions, but the effect size pooled estimates show a mix of very different PA-related outcome measures (steps, counts, distance, and intensity of PA) that, in most cases, are not interchangeable with each other. In accordance with these inconsistencies, a comprehensive quantitative analysis of the effects of different eHealth interventions aimed at promoting PA on PA and sedentary behaviors is lacking.

Consequently, this systematic review and meta-analysis aims to comprehensively analyze the available scientific literature on the effect of eHealth interventions promoting PA on PA parameters (count/min, steps/d, light PA [LPA], and MVPA) and sedentary behavior parameters (screen time and sedentary time) in children and adolescents and to clarify the potential reasons for the conflicting results. For this purpose, we will analyze the effect of eHealth interventions promoting PA compared with a control (waitlist, nontreatment, or minimal alternative intervention), hence analyzing the combination of intervention content (PA promotion) and intervention delivery mechanism (eHealth).

## Methods

### Systematic Review

This systematic review and meta-analysis has been reported according to the PRISMA (Preferred Reporting Items for Systematic Reviews and Meta-Analyses) guidelines [31] and the recommendations of the Cochrane Handbook for Systematic Reviews of Interventions [32] and was registered in PROSPERO (CRD42020211020), which has been modified to broaden the scope, changing the intervention scope (mHealth to eHealth) and the population (children to children and adolescents).

### Search Strategy

A systematic search of the literature was conducted in the CENTRAL, Embase (via Scopus), MEDLINE (via PubMed), and Web of Science databases from inception of the database to February 2022. The search strategy was designed using the PICO (patient, intervention, comparison, and outcomes) strategy, as shown in Textbox 1. The search strategy was designed combining the following relevant terms: (1) “m-health,” “ehealth,” “ICT,” “technology assisted,” “mobile technology,” “health technology,” “internet based,” “mobile health” and “mobile phone-based”; (2) “physical activity,” “exercise,” “fitness,” “cardiorespiratory fitness,” “aerobic fitness,” “physical fitness,” “step-count,” “daily steps,” “daily activity counts,” “sedentar*” and “screen-time”; (3) “effect,” “effecti*” and “evaluation”; and (4) “child*,” “infant,” “kids,” “young,” “adolescents.” Moreover, a reverse search was performed by checking the reference lists of previous systematic reviews and meta-analyses for other relevant studies. Moreover, previous systematic reviews [19-30] were reviewed to perform a reverse search. Detailed search strategies for each database are included in Table S1 of Multimedia Appendix 1 [33-57].

**Summary of the eligibility criteria following the PICO (patient, intervention, comparison, and outcomes) strategy.**

**Population**

- Inclusion: children and adolescents with mean age between 5 and 17 years without physical or psychological morbidities that would prevent the realization of the respective interventions
- Exclusion: studies including adult populations where disaggregation of data for children, adolescents, and adults was not possible

**Intervention**

- Inclusion: active monitoring or active interventions including eHealth technologies aimed to promote physical activity (PA) or reduce sedentary behaviors delivered to children and adolescents; multicomponent interventions including eHealth as the main component
- Exclusion: passive interventions (eg, nonfeedback monitoring), and multicomponent interventions not including eHealth as the main component or interventions aimed at physical exercise coaching

**Comparator**

- Inclusion: studies with waitlist, nonintervention, or usual care control groups
- Exclusion: studies without a control group or with a technology-delivered comparator

**Outcome**

- Inclusion: PA parameters (count/min, steps/d, light PA, and moderate to vigorous PA [MVPA]) and sedentary behavior parameters (screen time and sedentary time)
- Exclusion: eHealth intervention studies not involving PA or sedentary behavior parameters as a primary or secondary outcome

**Study design**

- Inclusion: experimental studies including randomized controlled trial (RCT) and feasibility studies with an RCT design
- Exclusion: nonexperimental study designs and non–peer-reviewed studies (eg, letters, comments, conference proceedings, reviews, and narrative articles)

### Selection of Studies

The eligibility criteria according to the PICO strategy are summarized in Textbox 1. For inclusion in this systematic review and meta-analysis, studies had to meet the following criteria: (1) designed as experimental studies (randomized controlled trials [RCTs] or feasibility studies with an RCT design); (2) interventions delivered to children and adolescents with a mean age between 5 and 17 years without physical or psychological morbidity that would prevent the respective interventions; (3) include active monitoring or active interventions with eHealth technologies as a main component aimed at promoting PA or reducing sedentary behaviors; (4) compared with a waitlist, no to minimal intervention, or usual care control group; and (5) report its effects on PA parameters (count/min, steps/d, LPA, and MVPA) and sedentary behavior parameters (screen time and sedentary time). The study population was limited to ages between 5 and 17 years to use homogeneous samples in physical and psychological development consistent with those used in different PA recommendations [9,10,58].

Studies were included when PA and sedentary behavior parameters were reported according to objective or subjective measures using established thresholds or cut points, not necessarily in the same units. When data were reported using both objective and subjective measures, the objective measures were prioritized. When trials reported multiple follow-up points, the longest available follow-up period was prioritized.

Studies were excluded if they were not written in English or Spanish or if they included adult populations where disaggregation of data for children, adolescents, and adults was not possible. Crossover studies were also excluded because of potential carryover effects resulting from a PA intervention that is not unreasonable to expect to continue for a prolonged period, which limited generalizability. Screening and trial selection were conducted independently by 2 reviewers (ISD and ICR), and disagreements were resolved by consensus.

### Data Extraction and Quality Assessment

The following information was extracted from the included studies: (1) study reference, (2) country, (3) study design, (4) population characteristics (sample size, percentage of female participants, mean age, and type of population), (5) outcome variables included, and (6) type of eHealth intervention.

The Cochrane Collaboration tool for assessing the Risk of Bias 2 (RoB2; version 2) [59] was used to assess the potential bias of the included RCTs. Five bias domains were reviewed: randomization process, deviations from intended interventions, missing outcome data, measurement of the outcome, and selection of the reported result. Studies could be rated as “low risk of bias” if all domains are classified as “low risk,” “some concerns” if there is at least 1 domain rated as “some concern,” and “high risk of bias” if there is at least 1 domain rated as “high risk” or ≥3 domains rated as “some concerns.” For cluster RCT, a specific version of the RoB2 tool with additional considerations was used [60], which includes a modification in the first domain to assess the identification or recruitment bias. A risk of bias (RoB) analysis was performed by analyzing the allocation to the intervention in both cases.

Data extraction and quality assessment were independently performed by 2 reviewers (ISD and ICR). Inconsistencies were resolved by consensus or by involving a third researcher (CPM).

### Grading the Quality of Evidence

The Grading of Recommendations Assessment, Development, and Evaluation (GRADE) framework was used to evaluate the certainty of evidence provided by this meta-analysis on the different outcomes [61]. Each outcome (counts/min, steps/d, LPA, MVPA, sedentary time, and screen time) could be scored as high, moderate, low, and very low evidence value, depending on the design of the studies, RoB, inconsistency, indirect evidence, imprecision, and publication bias. In this sense, some factors could increase or decrease the quality of the evidence score as follows: (1) RoB (−1 when <75% of the analyzed studies were at low RoB); (2) inconsistency (−1 when *I*^2^>50%); (3) indirect evidence related to indirect population, intervention, control, or outcomes; (4) imprecision related to wide CIs; and (5) publication bias (−1 when it exists).

### Statistical Analyses

When extracting data from the same trial, priority was given to the most recent and complete objectively measured data. For the statistical analysis, means before and after for both intervention and control groups (longest time after the intervention reported) with their respective SDs were preferably extracted. Data that did not meet these criteria as means before and after and SEs or mean differences (MDs) were extracted and converted. When data were reported as median (IQR), they were converted to mean (SD) following the most correct method according to the sample size devised by Hozo et al [62]. Furthermore, when primary data were reported in different units, unit conversions were performed.

To minimize *unit-of-analysis errors* when analyzing multiarm trials, similar intervention arms were combined into 1 arm. When interventions were not sufficiently similar, multiple entries were maintained in the splitting control group according to the number of intervention arms. Split samples were also combined.

To check for the normality of each outcome, we evaluated whether the authors tested the normality of the outcome through specific tests, and we calculated the mean/SD ratio for each intervention group (IG) to evaluate skewness.

When cluster RCTs were included, considering that the unit of allocation is the cluster, we checked for proper cluster analysis to avoid *unit-of-analysis error* [63]. When cluster RCTs were not appropriately analyzed, approximately correct analyses were performed using the inflated SEs method [64].

As opposed to final scores, change scores were analyzed, and when change was not available, final scores were converted to change scores using the following formula: *([pre-T mean – post-T mean] – [pre-C mean – post-C mean])*. When studies did not report change scores SDs and the exact correlation coefficients were not available, we assumed a correlation coefficient of 0.5 and calculated change scores SDs using Comprehensive Meta-Analysis software (version 2.2.064; Biostat, Inc.). The Hartung-Knapp-Sidik-Jonkman random effects method was used to compute the pooled MD estimates with their respective 95% CIs in PA parameters (counts/min, steps/d, LPA, and MVPA) and sedentary behavior parameters (sedentary time and screen time) [65]. Only the most recent analyses were included for quantitative synthesis when performed on the same population to avoid sample duplication. Moreover, when studies presented results stratified by sex, age, or weight status, they were analyzed as pooled.

The heterogeneity of results across studies was evaluated using the *I*^2^ statistic [30] considering the following values: might not be important (0%-30%), may represent moderate heterogeneity (30%-50%), substantial heterogeneity (50%-75%), or considerable heterogeneity (75%-100%). The corresponding *P* values were also taken into account [66]. Raw data and all data conversions are provided in the Multimedia Appendix 1.

Exploratory sensitivity analyses were performed to analyze the results when skewed data, high RoB data, and nonobjectively assessed data were removed. To assess the robustness of the pooled estimates and to detect whether any particular study accounted for a large proportion of heterogeneity, sensitivity analyses using the leave-one-out method were conducted. Furthermore, to analyze whether children with comorbidities could have influenced the results, an exploratory sensitivity analysis was performed.

Exploratory random effects meta-regression models were used to evaluate whether summary estimates were influenced by the percentage of female participants and the mean age of the participants. Exploratory multigroup analyses were performed based on the type of eHealth intervention (mobile phone app, multicomponent intervention, telephone-delivered intervention, text messages, or web-based intervention).

Finally, to evaluate publication bias, visual examination of funnel plots and the regression asymmetry test proposed by Egger [67] were used, and a *P* value <.10 was considered statistically significant.

Statistical analyses were performed using STATA SE software (version 15; StataCorp) and the *metagen* package from R statistical software (version 4.1.2; R Foundation for Statistical Computing).

## Results

### Systematic Review

**Figure 1:**
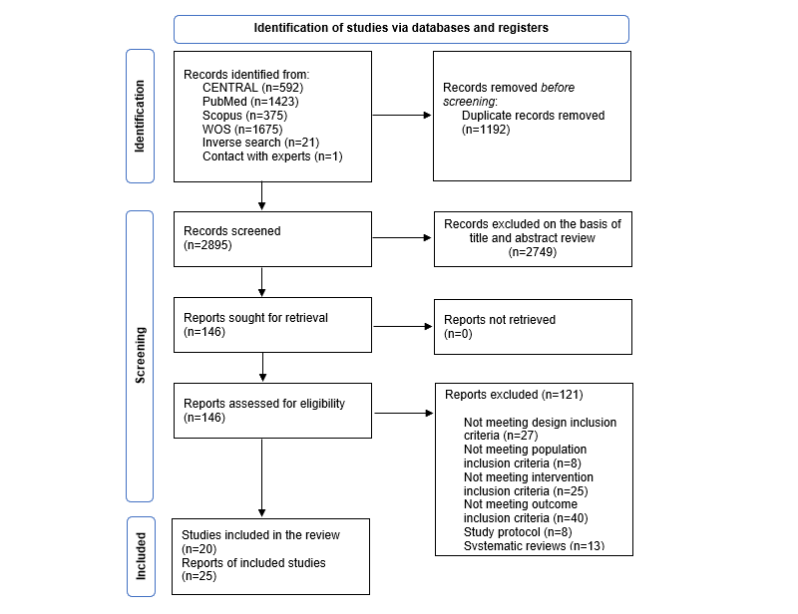
PRISMA (Preferred Reporting Items for Systematic Reviews and Meta-Analyses) diagram of the systematic literature search strategy. WOS: Web of Science.

The systematic search, which included an inverse search, retrieved a total of 4087 articles. One study that had not been retrieved through the systematic search was included by contact with experts [33]. After removing duplicates, 146 articles were selected based on the title and abstract screening for a full content review. Finally, 25 reports of 20 studies [33-57] met the inclusion criteria and were included in the systematic review (Figure 1). One study [68] was classified as “awaiting assessment” because more information was needed to clarify whether it was a duplicate report of 1 of the included studies [49]. Excluded studies and their respective reason for exclusion are displayed in Table S2 of Multimedia Appendix 1.

Table 1 summarizes the characteristics of the included studies. All studies meeting the inclusion criteria had an RCT design; 3 studies were pilot randomized trials [43,48,53], and 6 studies [36,37,41,50,52,54] had cluster RCT designs. Studies were published between 2006 and 2020 and were conducted in 7 different countries: Australia (3/20, 15%), Belgium (1/20, 5%), Finland (1/20, 5%), New Zealand (2/20, 10%), the Netherlands (3/20, 15%), the United Kingdom (1/20, 5%), and the United States (7/20, 35%), and 1 study [50] was conducted in 6 different European countries (Austria, Belgium, Crete, Germany, Greece, and Sweden).

**Table 1:** Characteristics of the included studies.

Study type and study name			Reference		Design		Country		Population characteristics							Outcome		
									Type of population		Participants, n (female)		Age (y), mean (SD)		PA^a^ parameters			Sedentary parameters
**App intervention**																		
	AIMFIT^b^	Direito et al [34], 2015		RCT^c^		New Zealand		Healthy adolescents		IG^d^1: 17 (9); IG2: 16 (10); CG^e^: 18 (10)		IG1: 15.78 (1.11); IG2: 15.69 (1.04); CG: 15.55 (1.32)		Counts per minute, PAQ-A^f^, PACES^g^, light PA, moderate PA, vigorous PA, and MVPA^h^ (min/d)			Sedentary time (min/d)	
**Exergame intervention**																		
	—^i^	Staiano et al [35], 2017		RCT		United States		Adolescents with overweight and obesity		IG: 19 (19); CG: 18 (18)		IG: 15.30 (1.3); CG: 16.10 (1.3)		Light PA, moderate PA, and vigorous PA (min/d)			Sedentary time (min/d)	
	GameSquad	Staiano et al [57], 2018		RCT		United States		Children with overweight and obesity		46 (21); IG: 23; CG: 23		11.2 (0.8)		MVPA (min/d)			—	
**Multicomponent intervention**																		
	Switch-off 4 Healthy Minds	Babic et al [36], 2016		Clustered RCT		Australia		Healthy adolescents		IG: 167 (107); CG: 155 (104)		IG: 14.47 (0.6); CG: 14.33 (0.5)		MVPA (min/d)			Screen time (min/d)	
	Nutrition and Enjoyable Activity for Teen Girls	Dewar et al [37], 2013 Dewar et al [38], 2014; Lubans et al [39], 2012		Clustered RCT		Australia		Healthy adolescents		IG: 178 (178) CG: 179 (179)		IG: 13.2 (0.5) CG: 13.2 (0.4)		Counts per minute and MVPA (min/d, %)			Screen time (min/d) and sedentary time (min/d)	
	Fit4Life	Huang et al [40], 2014		RCT		United States		Children surviving acute lymphoblastic leukemia		IG: 19 (7); CG: 19 (8)		IG: 13 (1.74); CG: 13 (1.74)		MVPA (min/d)			—	
	Avoiding Screen-time ATLAS^j^	Lubans et al [41], 2016; Smith et al [42], 2014		Clustered RCT		Australia		Healthy adolescents		IG: 181 (0); CG: 180 (0)		IG: 12.7 (0.5); CG: 12.7 (0.5)		Counts per minute and MVPA (%)			Screen time (min/d)	
	—	Mendoza et al [43], 2017		Pilot RCT		United States		Childhood cancer survivors		IG: 29 (17); CG: 30 (18)		IG: 16.9 (1.5); CG: 16.3 (1.5)		MVPA (min/d)			Sedentary time (min/d)	
	Pace-Internet for Diabetes Prevention Intervention (PACEi-DP)	Patrick et al [44], 2013		RCT		United States		Adolescents with overweight and obesity at risk of type 2 DM^k^		IG1 (W^l^): 26 (16); IG2 (WSMS^m^): 14 (12); IG3 (WG^n^): 26 (18); CG: 25 (18)		IG1 (W): 14.1 (1.4); IG2 (WSMS): 14.3 (1.8); IG3 (WG): 14.3 (1.5); CG: 14.5 (1.5)		MVPA (min/wk)			Sedentary time (h/d)	
	—	Ruotsalainen et al [33], 2015		RCT		Finland		Adolescents with overweight and obesity		IG1 (FB+Act^o^): 15 (10); IG2 (FB): 16 (11); CG: 15 (11)		IG1 (FB+Act): 14.8 (0.8); IG2 (FB): 14.8 (0.8); CG: 15 14.7 (0.8)		Light PA (min/d) and MVPA (min/d)			Sedentary time (min/d)	
	—	Thompson et al [45], 2016		RCT		United States		Healthy adolescents		IG: 40 (17); CG: 40 (20)		14 to 17		Steps/d and MVPA (min/d)			—	
**Telephone-delivered interventions**																		
	Healthy Eating and Activity Today study	Wright et al [46], 2013		RCT		United States		Children with obesity		IG: 24 (9); CG: 26 (12)		IG: 10.9 (1.3); CG: 10.5 (1.2)		—			Screen time (h/d)	
**Text messages intervention**																		
	—	Newton et al [47], 2009		RCT		New Zealand		Adolescents with type 1 DM		IG: 38 (22); CG: 40 (20)		14.4 (2.37)		Steps/d and MVPA (min/wk)			—	
	—	Shapiro et al [48], 2008		Pilot RCT		United States		Healthy adolescents		IG: 18 (13); CG: 22 (13)		IG: 8.4 (2.3); CG: 8.5 (2.3)		Steps/d			Screen time (min/d)	
**Web-based intervention**																		
	Rheumates@Work	Armbrust et al [49], 2017		RCT		The Netherlands		Children with juvenile idiopathic arthritis		IG: 28 (21); CG: 21 (12)		IG: 9.9 (0.78) CG: 10 (0.45)		Light PA (min/d) and MVPA (min/d)			—	
	Activ-O-Meter HELENA study	de Bourdeaudhuij et al [50], 2010; Cook et al [51], 2014		Clustered RCT		Europe		Healthy adolescents		1050 (515); IG: 581 (NR^p^); CG: 469 (NR)		14.5 (1.4)		MVPA (min/wk)			—	
	FATaintPHAT	Ezendam et al [52], 2012		Clustered RCT		The Netherlands		Healthy adolescents		IG: 485 (198); CG: 398 (200)		IG: 12.7 (0.7); CG: 12.6 (2.7)		Steps/d			Screen time (min/d)	
	Families Reporting Every Step to Health	Guagliano et al [53], 2020		Pilot RCT		United Kingdom		Healthy children		IG: 30 (15); CG: 29 (14)		IG: 10.1 (2.8); CG: 8.9 (0.6)		MVPA (min/d)			Sedentary time (min/d)	
	—	Haerens et al [54], 2006; Haerens et al [55], 2007		Clustered RCT		Belgium		Healthy children		IG1: 1194 (479); IG2: 911 (142); CG: 735 (432)		IG1: 13 (0.8); IG2: 13.2 (0.9); CG: 12.9 (0.7)		Light PA (min/d) and MVPA (min/d)			Sedentary time (min/d)	
	—	Slootmaker et al [56], 2010		RCT		The Netherlands		Healthy adolescents		IG: 41 (26); CG: 46 (29)		IG: 15.4 (1.1); CG: 14.9 (1.3)		Light PA (min/wk) and MVPA (min/wk)			Sedentary time (min/wk)	

^a^PA: physical activity.

^b^AIMFIT: Apps for Improving Fitness.

^c^RCT: randomized controlled trial.

^d^IG: intervention group.

^e^CG: control group.

^f^PAQ-A: Physical Activity Questionnaire for Adolescents.

^g^PACES: perceived enjoyment using the Physical Activity Enjoyment Scale.

^h^MVPA: moderate to vigorous physical activity.

^i^Not available.

^j^ATLAS: Active Teen Leaders Avoiding Screen-time.

^k^DM: diabetes mellitus.

^l^W: website only group.

^m^WSMS: website and SMS group.

^n^WG: website, monthly group sessions, and follow-up calls group.

^o^FB+Act: Facebook-delivered lifestyle counseling+physical activity self-monitoring group

^p^NR: not reported.

The sample size of the included studies ranged from 37 to 2840 participants (with a mean of 2436/4978, 58.7% female participants), whose ages ranged from 8.4 to 17 years; these studies were performed in children 6-12 years (5/20, 25%) and adolescents 13-17 years (15/20, 75%). Most studies included healthy children or adolescents (11/20, 55%), although several studies included children with physical health problems (overweight or obesity, 5/20, 25%; survivors of cancer, 2/20, 10%; and type 1 diabetes mellitus, 1/20, 5%).

Studies were classified according to the type of eHealth delivering the intervention (mobile apps, 1/20, 5%; exergames, 2/20, 10%; multicomponent interventions, 8/20, 40%; telephone-delivered interventions, 1/20, 5%; text messages, and 2/20, 10%; web-based interventions, 6/20, 30%). With regard to intervention content, most interventions were based on self-determination and social cognitive theories (5/20, 25% and 6/20, 30%, respectively), and included, in most cases, several of the following components: coaching (9/20, 45%), counseling (8/20, 40%), parental counseling (2/20, 10%), monitoring (2/20, 10%) and self-monitoring (3/20, 15%), group sessions (5/20, 25%), and goal setting (4/20, 20%). The duration of the interventions ranged from 8 to 96 weeks, and the dosage was left undetermined owing to the characteristics of the intervention. Most interventions required clinicians’ assistance (13/20, 65%); 20% (4/20) of the interventions were autonomous and 15% (3/20) of the interventions involved the school setting.

For the comparison, most studies used usual behavior (6/20, 30%), waitlist control (5/20, 25%), or no intervention (4/20, 20%). However, minimal interventions such as printed information, 1 group session, or generic advice were also used as control in 25% (5/20) of the studies.

Outcomes measured with objective measures such as accelerometers or pedometers were steps (4/20, 20%), counts (3/20, 15%), LPA (6/20, 30%), MVPA (13/20, 65%), sedentary time (6/20, 30%), and screen time (2/20, 10%). Several outcomes were also measured with self-reported instruments as questionnaires (screen time, 6/20, 30%; sedentary time, 2/20, 10%; or MVPA, 2/20, 10%).

Further details on intervention characteristics and outcome measurement methods are shown in Table S3 in Multimedia Appendix 1.

### RoB2 Tool

According to the Cochrane Collaboration’s tool for assessing the RoB, RoB2 for RCTs, 43% (6/14) of the trials showed a high RoB for overall bias, and 43% (6/14) the trials showed some concerns. By domain, no trial showed a high risk for the randomization process, missing outcome data, or selection of the reported result. Only 1 trial showed high risk for assignment to the intervention. The most compromised domain was measurement of the outcome, where 43% (6/14) showed a high RoB, probably owing to the nature of the interventions (Figure S1 in Multimedia Appendix 1).

Cluster RCTs RoB was assessed using a specific version of the RoB2 tool with additional considerations. According to the results, only 6% (1/16) of the trials showed a high RoB owing to a compromise in the measurement of the outcome domain, whereas the rest of the trials showed some concerns for the overall RoB (Figure S2 in Multimedia Appendix 1).

### Quality of Evidence

According to the GRADE summary of findings, the main limitations were the high RoB, the *substantial* heterogeneity, and imprecision among the RCTs included. Therefore, the level of certainty of the findings was very low for counts per minute, LPA, and sedentary time and low for steps per day, MVPA, and screen time (Table S4 in Multimedia Appendix 1).

### Meta-Analyses

When analyzing trials with several reports, the most recent and complete objectively measured data were considered for the quantitative synthesis. One study did not report sufficient numerical data to be included in the meta-analysis [57]. Only 1 of the included cluster RCTs [52] did not report an appropriate analysis to avoid “unit-of-analysis error” and did not report sufficient data to perform the inflated SEs method and was therefore excluded from the meta-analysis. Raw data and all conversions are reported in Multimedia Appendix 1.

To minimize the unit-of-analysis errors, studies with similar intervention arms were combined into 1 arm, as in the AIMFIT [34] study (combining IG1 and IG2), the PACEi-DP study [44] (combining IG1, IG2, and IG3), the studies by Haerens et al [54,55] combining IG1 and IG2, and the study by Slootmaker et al [56] combining boys and girls that had been analyzed separately.

None of the included studies reported change score SDs, and the exact correlation coefficients were only available for 1 study [49] for MVPA and LPA outcomes. However, because of the specific characteristics of the population included in this study, such coefficients were not used, and we assumed a correlation coefficient of 0.5 for the calculation of change score SDs.

**Figure 2:**
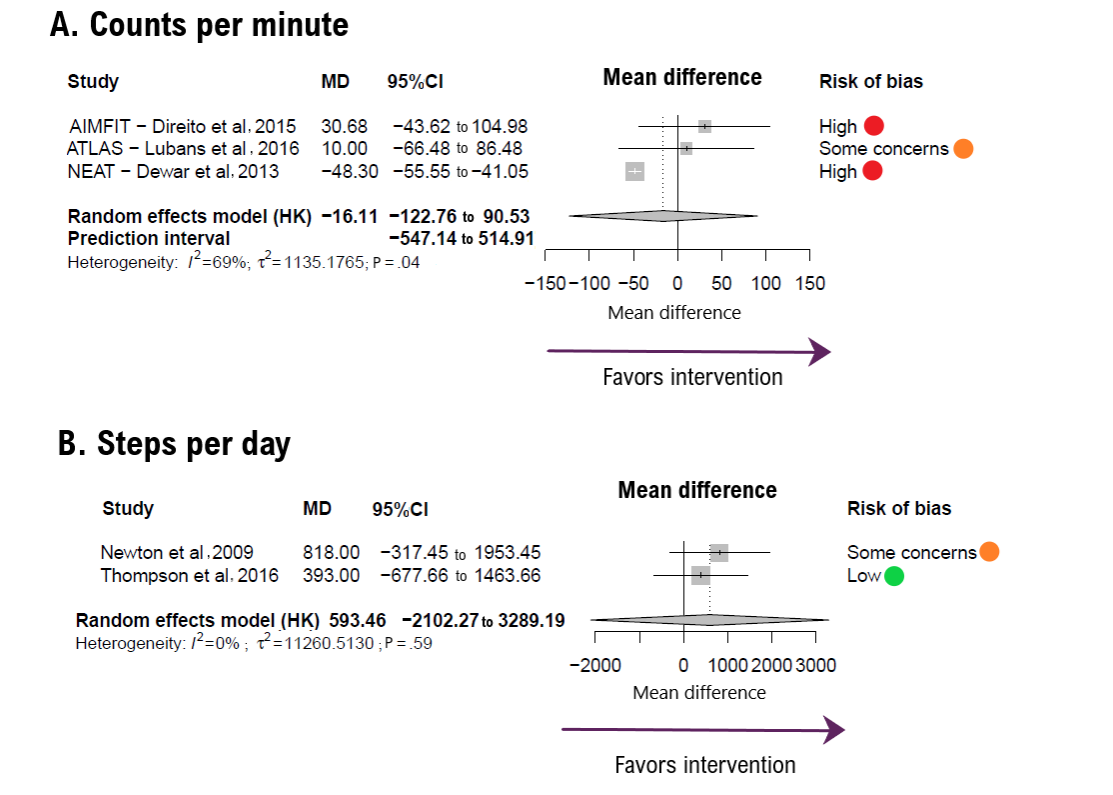
Forest plot showing the effect size for (A) counts per minute and (B) steps per day outcomes. Positive mean difference (MD) values favor intervention. Bold values highlight pooled effects (MD, 95% CI) and their respective prediction intervals. HK: Hartung-Knapp method.

The pooled MD for counts per minute (Figure 2 [34,37,41,45,47]) was −16.11 counts per minute (95% CI −122.76 to 90.53), with the number of trials (*k*=3) and number of participants (n=402) favoring control. For steps per day (Figure 2), MD was 593.46 steps (95% CI −2102.27 to 3289.19; *k*=2; n=152; favoring intervention [FI]). Both had substantial and insignificant heterogeneity values (*I*^2^=69% for counts/min *I*^2^=0%, for steps/d). The trial by Shapiro et al [48] was excluded from the steps per day analysis owing to its lack of a control group for this parameter.

**Figure 3:**
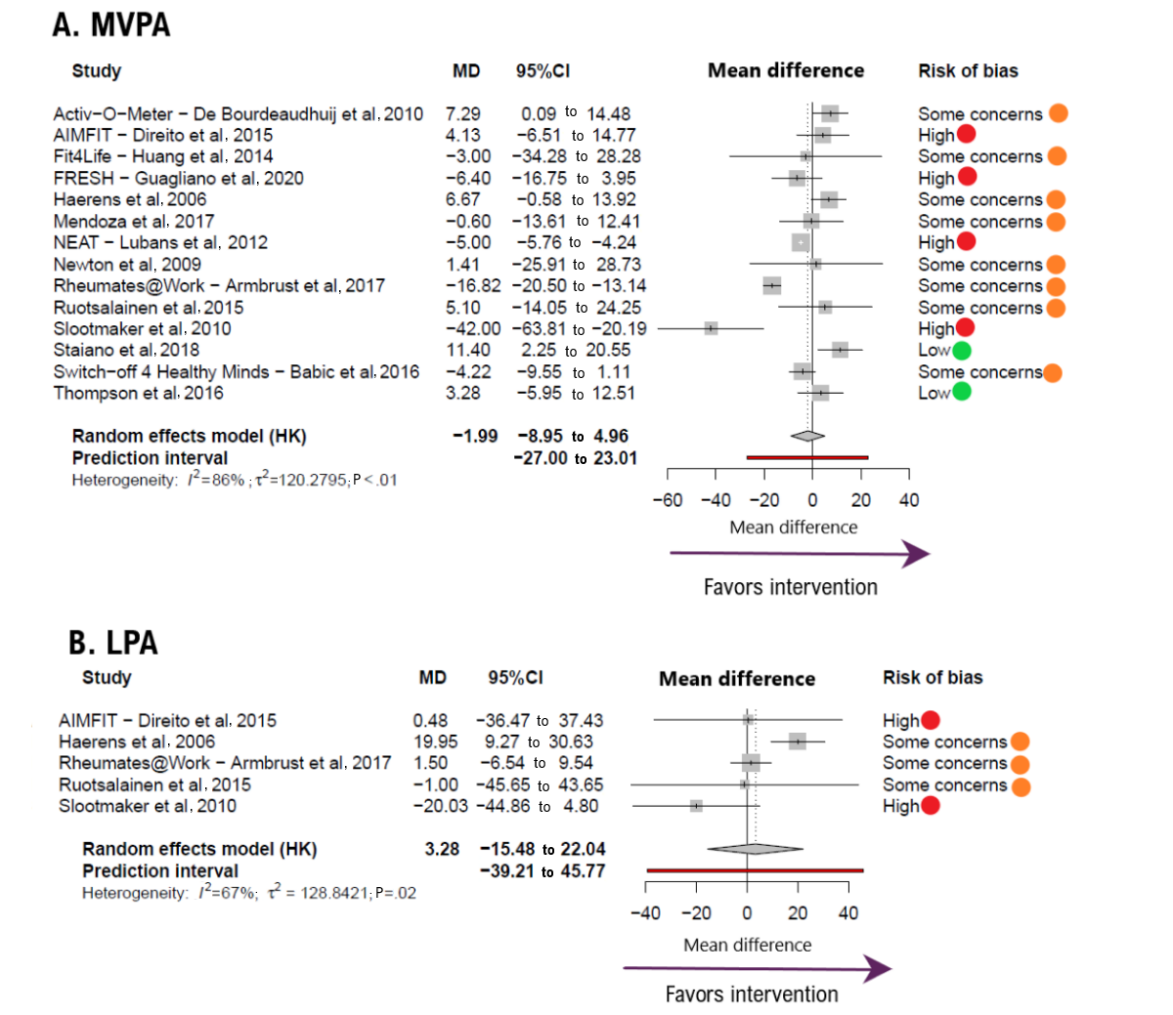
Forest plot showing the effect size for (A) moderate to vigorous physical activity (MVPA) and (B) light physical activity (LPA) outcomes. Positive mean difference (MD) values favor intervention. Bold values highlight pooled effects (MD, 95% CI) and their respective prediction intervals.

The pooled MD for MVPA (Figure 3 [33,34,36,39,40,43, 45,47,49,50,53,54,56,57]) was −1.99 minute per day (95% CI −8.95 to 4.96; *k*=14; n=2336; favoring control). After extracting data for the analysis, 1 study [43] showed log transformed data for MVPA and was consequently excluded from the analysis. For LPA (Figure 3), MD was 3.28 minute per day (95% CI −15.48 to 22.04; *k*=5; n=355; FI). The heterogeneity values were substantial to considerable (*I*^2^=86% for MVPA and *I*^2^=67%, for LPA).

**Figure 4:**
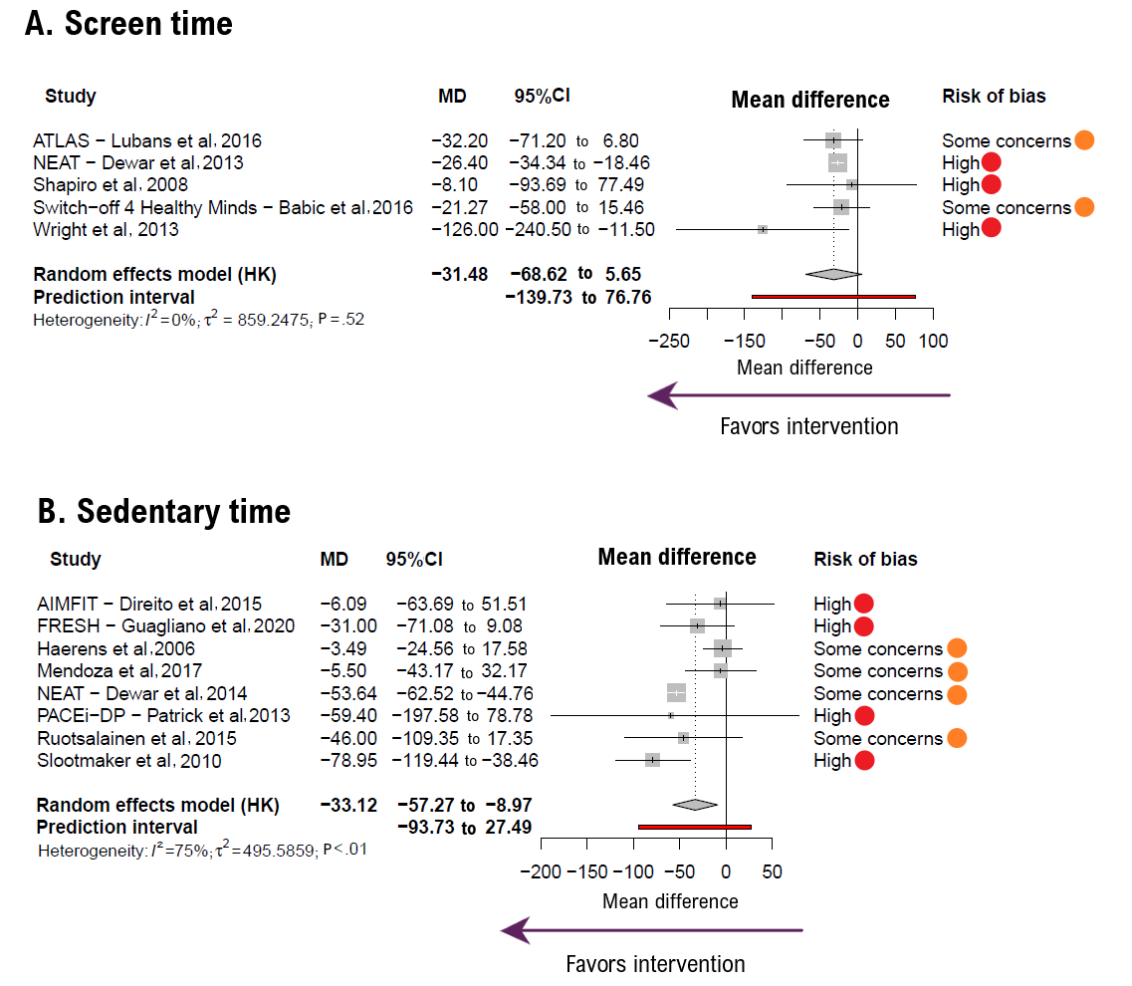
Forest plot showing the effect size for (A) screen time and (B) sedentary time outcomes. Negative mean difference (MD) values favor intervention. Bold values highlight pooled effects (MD, 95% CI) and their respective prediction intervals. HK: Hartung-Knapp method.

For sedentary behavior parameters, the pooled MD for screen time (Figure 4 [33,34,36-38,41,43,44,46,48,53,54,56]) was −31.48 minute per day (95% CI −68.62 to 5.65; *k*=5; n=904, FI). For sedentary time (Figure 3), the MD was −33.12 minute per day (95% CI −57.27 to −8.97; *k*=8; n=819; FI). There were no substantial heterogeneity estimates (*I*^2^=0% for screen time and *I*^2^=75%, for sedentary time).

### Sensitivity Analysis

After evaluating skewness to check for normality of primary data, evidence of skewness was found for several reports in steps per day, MVPA, screen time, and sedentary time analyses, and taking into account the nature of the data, a sensitivity analysis excluding skewed data was performed. Such analysis showed no significant statistical differences from the main analyses, except for the loss of statistical significance in the sedentary time MD (Table 2).

**Table 2:** Sensitivity analysis results.

Outcome			Number of trials	Pooled MD^a^ estimates (95% CI)		*I*^2^ (%)
**Pooled MD estimates excluding skewed data**						
	Steps per day	*k*=1 [44]		393 (−677.66 to 1463.66)	—^b^	
	MVPA^c^ (min/d)	*k*=3 [38,48,56]		−18.89 (−63.09 to 25.32)	95.9	
	Screen time (min/d)	*k*=1 [36]		−*48.30 (−55.55 to −41.05)*^d^	—	
	Sedentary time (min/d)	*k*=4 [33,37,54,56]		−36.69 (−93.97 to 20.58)	86.9	
**Pooled MD estimates excluding high risk of bias data**						
	Counts per minute	*k*=1 [40]		10 (−66.48 to 86.48)	—	
	MVPA (min/d)	*k*=10 [34,35,39,42,44,48,50,54,56,67]		0.54 (−6.07 to 7.14)	88.4	
	LPA^e^ (min/d)	*k*=3 [48,54,67]		9.27 (−19.10 to 37.65)	73.3	
	Screen time (min/d)	*k*=2 [35,40]		−26.41 (−95.73 to 42.91)	0.0	
	Sedentary time (min/d)	*k*=4 [37,42,54,67]		−27.08 (−70.79 to 16.63)	86.8	
**Pooled MD estimates excluding nonobjectively measured data**						
	MVPA (min/d)	*k*=11 [33-35,38,39,42,44,48,53,54,67]		−1.33 (−7.02 to 4.35)	85.6	
	LPA (min/d)	*k*=4 [33,48,54,67]		8.57 (−8.37 to 25.51)	60.7	
	Screen time	*k*=0		—	—	
	Sedentary time (min/d)	*k*=6 [33,37,42,53,54,67]		−*26.45 (−51.80 to −1.10)*	79.8	
**Pooled MD estimates excluding converted data**						
	Counts per minute	*k*=1 [33]		10.00 (−66.48 to 86.48)	—	
	Steps per day	*k*=0		—	—	
	MVPA (min/d)	*k*=6 [33,34,39,50,53,67]		3.66 (−2.17 to 9.50)	7.3	
	LPA (min/d)	*k*=3 [33,54,67]		14.94 (−11.52 to 41.40)	0.0	
	Screen time (min/d)	*k*=2 [45,47]		−60.32 (−804.46 to 683.81)	61.7	
	Sedentary time (min/d)	*k*=4 [33,53,54,67]		−15.23 (−44.22 to 13.75)	0.0	
**Pooled MD estimates including only healthy population**						
	Steps per day	*k*=1 [44]		393 (−677.66 to 1463.66)	—	
	MVPA (min/d)	*k*=8 [33,35,38,44,50,53,54,56]		−2.86 (−14.45 to 8.74)	81.4	
	LPA (min/d)	*k*=3 [33,54,56]		2.66 (−50.21 to 55.54)	77.4	
	Screen time (min/d)	*k*=4 [35,36,40,47]		−*26.20 (−31.06 to −21.34)*	0.0	
	Sedentary time (min/d)	*k*=5 [33,37,53,54,56]		−35.81 (−74.77 to 3.14)	82.9	

^a^MD: mean difference.

^b^Not available.

^c^MVPA: moderate to vigorous physical activity.

^d^Italicized values indicate statistical significance (*P*≤.05).

^e^LPA: light physical activity.

In addition, exploratory sensitivity analyses were conducted, excluding trials classified as high RoB and those using nonobjective measurement methods. These results did not significantly differ from the main analyses but differed for sedentary time (Table 2).

Furthermore, owing to the different formats of primary data, an exploratory sensitivity analysis was performed by analyzing only primary data reported as means (SD), which did not significantly differ from the main analysis for the loss of statistical significance in the sedentary time MD (Table 2).

Moreover, studies were removed one at a time from each analysis to examine their individual impact on pooled MD estimates, which were not significantly modified in magnitude or direction when each trial was removed in any of the outcomes.

When performing an exploratory sensitivity analysis including only children and adolescents reported as healthy, only screen time was statistically significantly modified (*k*=4; –26.20 min/d, 95% CI –31.06 to –21.34).

### Meta-Regression and Multiple Group Analysis

Exploratory random effects meta-regression models showed that neither the age of the participants nor the percentage of female participants included in the samples could have influenced the pooled standardized MD estimates for any of the outcomes studied (Table S5 in Multimedia Appendix 1).

Table S6 in Multimedia Appendix 1 shows the results of exploratory multigroup analyses, which, although there were not enough studies to have a meaningful impact on our results, were included along with tests for subgroup differences to show how evidence was distributed between the different types of eHealth interventions. Furthermore, it is important to note that these analyses are derived from observations and lack sufficient statistical power.

### Publication Bias

After visually examining the funnel plots (Figures S3-S8 in Multimedia Appendix 1) and performing the Egger tests for each parameter (Table S7 in Multimedia Appendix 1), no evidence of significant publication bias was found.

## Discussion

### Principal Findings

This systematic review and meta-analysis provides a quantitative analysis of the effects of eHealth interventions on the different parameters of PA and sedentary behavior in children and adolescents. Overall, our results showed that eHealth interventions aimed at increasing PA and decreasing time spent in sedentary behaviors in children did not have a significant effect on increasing PA or decreasing screen time. However, they showed a statistically significant reduction in time spent on sedentary behavior that was not robust to any sensitivity analysis, highlighting the need for further research to confirm these findings.

According to our results, eHealth interventions aimed at promoting PA showed a greater effect on reducing some sedentary behavior parameters, such as time spent in sedentary behaviors or screen time, than on increasing PA, especially MVPA. These findings are in line with the results of previous systematic reviews and meta-analyses [21-23], and considering the importance of PA, especially MVPA for a healthy development [10], the lack of effect of increasing MVPA highlights one of the main limitations of this type of intervention. However, further research is required to elucidate whether more specific interventions or modified interventions could improve MVPA.

The statistically significant reduction in sedentary time, which has not been shown previously when analyzing only mHealth interventions [26], is particularly important because sedentary behaviors, especially screen-associated sedentary behaviors such as screen time, have been recognized as significant contributors to adverse health [69]. Moreover, eHealth interventions have been previously questioned owing to their potentially adverse consequences of increased sedentary screen time and decreased focused attention owing to technology use [70]; hence, our results suggest the possibility of benefits outweighing potential harm. Although it is true that our estimates are based on data from only a few studies and are not robust to sensitivity analyses performed, they need to be confirmed by subsequent studies.

Although the results of previous meta-analyses in older people raise expectations about the usefulness of eHealth interventions for increasing PA [71-73], the results in children have not shown strong beneficial effects on daily waking activities [19-30]. Our findings are in line with previous systematic reviews and meta-analyses [21-23,25-27], as the heterogeneity of the designs, small sample sizes, and variety of outcomes and devices analyzed suggest that further research is needed. This is particularly important given the rapid growth of eHealth technologies, which, considering the high replacement rate, makes studies obsolete in a short period and consequently challenges research to keep pace [74].

Several reasons could be hypothesized to explain these contradictory differences in the effect of eHealth technologies on children and older people, including the following: (1) most interventions in children lack a behavioral change model, or the model is inappropriate; (2) because children are “born connected,” they are assumed to have a literacy in the use of these technologies that may be lacking or may not be sufficient for appropriate use; (3) the use of these technologies may require a change in children’s daily routines, thus requiring parental involvement; and (4) owing to intervention strategies being unable to meet children’s needs, it may be difficult to achieve and maintain motivation and benefit perception to preserve adherence.

In view of these results, should we discard any hope that such interventions are effective in promoting PA? It seems logical that to answer this question, studies must first be conducted to overcome the aforementioned shortcomings (small number of studies, small sample sizes, high heterogeneity, and poor quality) of the studies conducted to date.

eHealth interventions promoting PA, despite controversies about their effectiveness, are described as acceptable, usable, and feasible, with benefits such as cost-effectiveness, potential for real-time data collection, feedback capability, minimized participant burden, and increased dissemination capability [70]. Thus, they are postulated as promising alternatives to truly scalable interventions; however, such benefits must be confirmed through studies involving larger sample sizes. Hence, future research should focus on developing eHealth interventions adapted to the pediatric population, which could be tested in large RCTs with a clearer and comparable methodology.

### Limitations

Our results should be interpreted cautiously, as our study has some limitations that should be acknowledged. First, the paucity of comparable RCTs, RCTs registered but not yet published, or gray literature that may have been overlooked in our systematic search may have affected the magnitude of our estimates. Second, the heterogeneity of some of the included interventions owing to differences in their components, length, main outcomes, or measurement instruments may also have affected our findings. Third, the small sample size of some of the included studies decreased the precision of our estimates. Fourth, challenges arise in estimating the independent effect of each component when analyzing multicomponent interventions or interventions whose primary objective is not promoting PA. Fifth, approximately 50% (6/14) of the included trials showed a high RoB, which could compromise the consistency of our findings. Sixth, several exploratory multigroup analyses were performed, even knowing that the Cochrane Handbook for Systematic Reviews of Interventions [32] recommends that these analyses should include at least 10 studies for the same outcome, recognizing the threat it poses to the inferences based on our estimates. Finally, none of the included studies used the mHealth evidence reporting and assessment checklist [75].

### Conclusions

Our results provide a comprehensive quantitative analysis of the effects of eHealth interventions on PA and sedentary behavior parameters in children and adolescents according to PRISMA standards (Multimedia Appendix 2). We did not find evidence of an effect of PA-targeted eHealth interventions on PA parameters, but the very low certainty of evidence suggests that eHealth interventions may reduce sedentary time in children and adolescents. Our findings may have an important scientific impact as they highlight that fast-paced advancements in eHealth interventions aimed at promoting PA lack robust supporting evidence.

## Appendix

Multimedia Appendix 1 Supplementary material including in-depth step-by-step analyses.

Multimedia Appendix 2 PRISMA (Preferred Reporting Items for Systematic Reviews and Meta-Analyses) checklist.

## Abbreviations

FI: favoring intervention
GRADE: Grading of Recommendations Assessment, Development, and Evaluation
IG: intervention group
LPA: light physical activity
MD: mean difference
mHealth: mobile health
MVPA: moderate to vigorous physical activity
PA: physical activity
PICO: patient, intervention, comparison, and outcomes
PRISMA: Preferred Reporting Items for Systematic Reviews and Meta-Analyses
RCT: randomized controlled trial
RoB: risk of bias
RoB2: Risk of Bias 2
