# Supplementary material for: Effectiveness of eHealth Interventions Promoting Physical Activity in Children and Adolescents: Systematic Review and Meta-Analysis
Source: J Med Internet Res. 2024 Feb 21;26:e41649. doi: 10.2196/41649 (PMC10918549; doi:10.2196/41649)
Supplement: Multimedia Appendix 1 [file jmir_v26i1e41649_app1.docx]

**SUPPLEMENTARY MATERIAL**

**Table S1**. Search strategy for each database

|  |  |  | eTable 1. Search strategy for each database.  eTable 1. Search strategy for each database. |
| --- | --- | --- | --- |
| \| **Date:** February 2022**.** \| \| --- \| \| **MEDLINE (via Pubmed)** \| \| (("mhealth" OR "ehealth" OR ICT OR “technology assisted” OR “mobile technology” OR “health technology” OR “internet based” OR “mobile health” OR “mobile phone-based”) AND ("physical activity" OR exercise OR “fitness” OR “cardiorespiratory fitness” OR “aerobic fitness” OR “physical fitness” OR “step-count” OR “daily steps” OR “daily activity counts” OR sleep OR sedentar* OR “screen time”) AND (effect OR effecti* OR evaluation) AND (child* OR infant OR kids OR young OR adolescents))  **Results: 1423** \| \| **Scopus** \| \| **TITLE-ABS** ( ( "mhealth" OR "ehealth" OR ict OR "technology assisted" OR "mobile technology" OR "health technology" OR "internet based" OR "mobile health" OR "mobile phone-based" ) AND ( "physical activity" OR exercise OR "fitness" OR "cardiorespiratory fitness" OR "aerobic fitness" OR "physical fitness" OR "step-count" OR "daily steps" OR "daily activity counts" OR sleep OR sedentar* OR "screen time" ) AND ( effect OR effecti* OR evaluation ) AND ( child* OR infant OR kids OR young OR adolescents ))  **Results: 375** \| \| **Web Of Science** \| \| TS=(("mealth" OR "eheat" OR ICT OR “technology assisted” OR “mobile technology” OR “health technology” OR “internet based” OR “mobile health” OR “mobile phone-based”) AND ("physical activity" OR exercise OR “fitness” OR “cardiorespiratory fitness” OR “aerobic fitness” OR “physical fitness” OR “step-count” OR “daily steps” OR “daily activity counts” OR sleep OR sedentar* OR “screen time”) AND (effect OR effecti* OR evaluation) AND (child* OR infant OR kids OR young OR adolescents) )  **Results: 1675** \| \| **Cochrane Central Register of Controlled Trials** \| \| (("mhealth" OR "ehealth" OR ICT OR “technology assisted” OR “mobile technology” OR “health technology” OR “internet based” OR “mobile health” OR “mobile phone-based”) AND ("physical activity" OR exercise OR “fitness” OR “cardiorespiratory fitness” OR “aerobic fitness” OR “physical fitness” OR “step-count” OR “daily steps” OR “daily activity counts” OR sleep OR sedentar* OR “screen time”) AND (effect OR effecti* OR evaluation) AND (child* OR infant OR kids OR young OR adolescents))  **Results: 592** \| \| **TOTAL results: 4065**  **Results after duplicated removed: 2895** (with Mendeley desktop find and merge duplicated tool). \| |  |  |  |
|  |  |  |  |

**Table S2**. Excluded studies by reason for exclusion (n = 121).

| **Not meeting design inclusion criteria (n =27)** |
| --- |
| 1. Benavides, C., Benítez-Andrades, J. A., Marqués-Sánchez, P., & Arias, N. eHealth intervention to improve health habits in the adolescent population: mixed methods study. *JMIR mHealth and uHealth* 2021, 9(2), e20217. |
| 1. Benítez-Andrades, J. A., Arias, N., García-Ordás, M. T., Martínez-Martínez, M., & García-Rodríguez, I. Feasibility of social-network-based eHealth intervention on the improvement of healthy habits among children. *Sensors* 2020, 20(5), 1404. |
| 1. Bexelius, C., Sandin, S., Trolle Lagerros, Y., Litton, J. E., & Löf, M. Estimation of physical activity levels using cell phone questionnaires: a comparison with accelerometry for evaluation of between-subject and within-subject variations. *Journal of medical internet research* 2011, 13(3), e70. |
| 1. Brannon, E. E., Cushing, C. C., Walters, R. W., Crick, C., Noser, A. E., & Mullins, L. L. Goal feedback from whom? A physical activity intervention using an N-of-1 RCT. *Psychology & health* 2018, 33(6), 701-712. |
| 1. Bronikowski, M., Bronikowska, M., & Glapa, A. Do they need goals or support? A report from a goal-setting intervention using physical activity monitors in youth. *International journal of environmental research and public health* 2016, 13(9), 914. |
| 1. Chew, C. S. E., Davis, C., Lim, J. K. E., Lim, C. M. M., Tan, Y. Z. H., Oh, J. Y., ... & Finkelstein, E. A. Use of a mobile lifestyle intervention app as an early intervention for adolescents with obesity: Single-cohort study. *Journal of Medical Internet Research* 2021, 23(9), e20520. |
| 1. Cueto, V., Wang, C. J., & Sanders, L. M. Impact of a mobile app–based health coaching and behavior change program on participant engagement and weight status of overweight and obese children: Retrospective cohort study. *JMIR mHealth and uHealth* 2019, 7(11), e14458. |
| 1. Cushing, C. C., Mitchell, T. B., Bejarano, C. M., Walters, R. W., Crick, C. J., & Noser, A. E. Bidirectional associations between psychological states and physical activity in adolescents: A mHealth pilot study. *Journal of pediatric psychology* 2017, 42(5), 559-568. |
| 1. Degroote, L., De Paepe, A., De Bourdeaudhuij, I., Van Dyck, D., & Crombez, G. Effectiveness of the mHealth intervention ‘MyDayPlan’to increase physical activity: an aggregated single case approach. *International Journal of Behavioral Nutrition and Physical Activity* 2021, 18(1), 1-12. |
| 1. Dunton, G. F., Liao, Y., Intille, S. S., Spruijt‐Metz, D., & Pentz, M. Investigating children's physical activity and sedentary behavior using ecological momentary assessment with mobile phones. *Obesity* 2011, 19(6), 1205-1212. |
| 1. Ermetici, F., Zelaschi, R. F., Briganti, S., Dozio, E., Gaeta, M., Ambrogi, F., ... & Malavazos, A. E. Association between a school‐based intervention and adiposity outcomes in adolescents: The Italian “EAT” project. Obesity 2016, 24(3), 687-695. |
| 1. Galy, O., Yacef, K., & Caillaud, C. Improving pacific adolescents’ physical activity toward international recommendations: Exploratory study of a digital education app coupled with activity trackers. *JMIR mHealth and uHealth* 2019, 7(12), e14854. |
| 1. Garde, A., Umedaly, A., Abulnaga, S. M., Junker, A., Chanoine, J. P., Johnson, M., ... & Dumont, G. A. Evaluation of a novel mobile exergame in a school-based environment. *Cyberpsychology, Behavior, and Social Networking* 2016, 19(3), 186-192. |
| 1. Garde, A., Umedaly, A., Abulnaga, S. M., Robertson, L., Junker, A., Chanoine, J. P., ... & Dumont, G. A. Assessment of a mobile game (“MobileKids Monster Manor”) to promote physical activity among children. *Games for health journal* 2015, 4(2), 149-158. |
| 1. Garde, A., Chowdhury, M., Rollinson, A. U., Johnson, M., Prescod, P., Chanoine, J. P., ... & Dumont, G. A. A multi-week assessment of a mobile exergame intervention in an elementary school. *Games for Health Journal* 2018, 7(1), 43-50. |
| 1. Gaudet, J., Gallant, F., & Bélanger, M. A bit of fit: minimalist intervention in adolescents based on a physical activity tracker. *JMIR mHealth and uHealth* 2017, 5(7), e7647. |
| 1. Hayes, L. B., & Van Camp, C. M. Increasing physical activity of children during school recess. *Journal of applied behavior analysis* 2015, 48(3), 690-695. |
| 1. Hooke, M. C., Gilchrist, L., Tanner, L., Hart, N., & Withycombe, J. S. Use of a fitness tracker to promote physical activity in children with acute lymphoblastic leukemia. *Pediatric blood & cancer* 2016, 63(4), 684-689. |
| 1. Patten, J. W., Iarocci, G., & Bojin, N. A pilot study of children’s physical activity levels during imagination-based mobile games. *Journal of Child Health Care* 2017, 21(3), 292-300. |
| 1. Pyky, R., Koivumaa-Honkanen, H., Leinonen, A. M., Ahola, R., Hirvonen, N., Enwald, H., ... & Korpelainen, R. Effect of tailored, gamified, mobile physical activity intervention on life satisfaction and self-rated health in young adolescent men: A population-based, randomized controlled trial (MOPO study). *Computers in Human Behavior* 2017, 72, 13-22. |
| 1. Schaefer, S. E., Ching, C. C., Breen, H., & German, J. B. Wearing, thinking, and moving: testing the feasibility of fitness tracking with urban youth. American Journal of Health Education 2016, 47(1), 8-16. |
| 1. Schaefer, S. E., Van Loan, M., & German, J. B. Peer reviewed: A feasibility study of wearable activity monitors for pre-adolescent school-age children. Preventing chronic disease 2014, 11. |
| 1. Sousa, P., Fonseca, H., Gaspar, P., & Gaspar, F. Usability of an internet-based platform (Next. Step) for adolescent weight management. *Jornal de pediatria* 2015, 91, 68-74. |
| 1. Thorén, A., Janson, A., Englund, E., & Silfverdal, S. A. Development, implementation and early results of a 12‐week web‐based intervention targeting 51 children age 5–13 years and their families. Obesity science & practice 2020, 6(5), 516-523. |
| 1. Williamson, D. A., Walden, H. M., White, M. A., York‐Crowe, E., Newton Jr, R. L., Alfonso, A., ... & Ryan, D. Two‐year internet‐based randomized controlled trial for weight loss in African‐American girls. *Obesity* 2006, 14(7), 1231-1243. |
| 1. Wilson, M., Ramsay, S., & Young, K. J. Engaging overweight adolescents in a health and fitness program using wearable activity trackers. *Journal of Pediatric Health Care* 2017, 31(4), e25-e34. |
| 1. Ye, S., Pope, Z. C., Lee, J. E., & Gao, Z. Effects of school-based exergaming on urban children’s physical activity and cardiorespiratory fitness: A quasi-experimental study. *International journal of environmental research and public health* 2019, 16(21), 4080. |
| **Not meeting population inclusion criteria (n=8)** |
| 1. Al-Nawaiseh, H. K., McIntosh, W. A., & McKyer, L. J. An-m-health intervention using smartphone app to improve physical activity in college students: a randomized controlled trial. *International Journal of Environmental Research and Public Health* 2022, 19(12), 7228. |
| 1. Delisle Nyström, C., Sandin, S., Henriksson, P., Henriksson, H., Trolle-Lagerros, Y., Larsson, C., ... & Löf, M. Mobile-based intervention intended to stop obesity in preschool-aged children: the MINISTOP randomized controlled trial. *The American journal of clinical nutrition* 2017, 105(6), 1327-1335. |
| 1. Delisle Nyström, C., Sandin, S., Henriksson, P., Henriksson, H., Maddison, R., & Löf, M. A 12-month follow-up of a mobile-based (mHealth) obesity prevention intervention in pre-school children: the MINISTOP randomized controlled trial. *BMC Public Health* 2018, 18(1), 1-7. |
| 1. Downing, K. L., Salmon, J., Hinkley, T., Hnatiuk, J. A., & Hesketh, K. D. Feasibility and efficacy of a parent-focused, text message–delivered intervention to reduce sedentary behavior in 2-to 4-year-old children (Mini movers): pilot randomized controlled trial. JMIR mHealth and uHealth 2018, 6(2), e8573. |
| 1. Hammersley, M. L., Okely, A. D., Batterham, M. J., & Jones, R. A. An internet-based childhood obesity prevention program (Time2bHealthy) for parents of preschool-aged children: randomized controlled trial. *Journal of medical Internet research* 2019, 21(2), e11964. |
| 1. Hammersley, M. L., Wyse, R. J., Jones, R. A., Okely, A. D., Wolfenden, L., Eckermann, S., ... & Rissel, C. Telephone and Web-Based Delivery of Healthy Eating and Active Living Interventions for Parents of Children Aged 2 to 6 Years: Mixed Methods Process Evaluation of the Time for Healthy Habits Translation Trial. *Journal of medical Internet research* 2022, 24(5), e35771. |
| 1. Miragall, M., Domínguez-Rodríguez, A., Navarro, J., Cebolla, A., & Baños, R. M. Increasing physical activity through an Internet-based motivational intervention supported by pedometers in a sample of sedentary students: A randomised controlled trial. *Psychology & health* 2018, 33(4), 465-482. |
| 1. Nyström, M. B., Stenling, A., Sjöström, E., Neely, G., Lindner, P., Hassmén, P., ... & Carlbring, P. Behavioral activation versus physical activity via the internet: A randomized controlled trial. *Journal of affective disorders* 2017, 215, 85-93. |
| **Not meeting intervention inclusion criteria (n =25)** |
| 1. Adab, P., Barrett, T., Bhopal, R., Cade, J. E., Canaway, A., Cheng, K. K., ... & Passmore, S. The West Midlands ActiVe lifestyle and healthy Eating in School children (WAVES) study: a cluster randomised controlled trial testing the clinical effectiveness and cost-effectiveness of a multifaceted obesity prevention intervention programme targeted at children aged 6-7 years. *Health Technology Assessment* 2018, 22(8), 1. |
| 1. Aittasalo, M., Jussila, A. M., Tokola, K., Sievänen, H., Vähä-Ypyä, H., & Vasankari, T. Kids Out; evaluation of a brief multimodal cluster randomized intervention integrated in health education lessons to increase physical activity and reduce sedentary behavior among eighth graders. BMC public health 2019, 19, 1-16. |
| 1. Appel, H. B., Huang, B., Cole, A., James, R., & Ai, A. L. Starting the conversation-a childhood obesity knowledge project using an app. British journal of medicine and medical research 2014, 4(7), 1526-1538. |
| 1. Catenacci, V., Barrett, C., Odgen, L., Browning, R., Schaefer, C. A., Hill, J., & Wyatt, H. Changes in physical activity and sedentary behavior in a randomized trial of an internet-based versus workbook-based family intervention study. *Journal of Physical Activity and Health*, 2014 11(2), 348-358. |
| 1. Chen J, Guedes C, Cooper B, Lung A., Short-Term Efficacy of an Innovative Mobile Phone Technology-Based Intervention for Weight Management for Overweight and Obese Adolescents: Pilot Study *Interact J Med Res* 2017;6(2):e12 |
| 1. Chen, J. L., Weiss, S., Heyman, M. B., Cooper, B., & Lustig, R. H. The efficacy of the web-based childhood obesity prevention program in Chinese American adolescents (Web ABC study). *Journal of Adolescent Health* 2011, 49(2), 148-154. |
| 1. Davis, A. M., Sampilo, M., Gallagher, K. S., Landrum, Y., & Malone, B. Treating rural pediatric obesity through telemedicine: outcomes from a small randomized controlled trial. *Journal of pediatric psychology* 2013, 38(9), 932-943. |
| 1. Frenn, M., Malin, S., Brown, R. L., Greer, Y., Fox, J., Greer, J., & Smyczek, S. Changing the tide: an Internet/video exercise and low-fat diet intervention with middle-school students. *Applied nursing research* 2005, 18(1), 13-21. |
| 1. Gorely T, Nevill ME, Morris JG, Stensel DJ, Nevill A. Effect of a school-based intervention to promote healthy lifestyles in 7-11 year old children. Int J Behav Nutr Phys Act. 2009 Jan 21;6:5. |
| 1. Horne, P. J., Hardman, C. A., Lowe, C. F., & Rowlands, A. V. Increasing children's physical activity: a peer modelling, rewards and pedometer-based intervention. European Journal of clinical nutrition 2009, 63(2), 191-198. |
| 1. Jago, R., Baranowski, T., Baranowski, J. C., Thompson, D., Cullen, K. W., Watson, K., & Liu, Y. Fit for Life Boy Scout badge: outcome evaluation of a troop and Internet intervention. *Preventive medicine* 2006, 42(3), 181-187. |
| 1. Jones, M., Taylor Lynch, K., Kass, A. E., Burrows, A., Williams, J., Wilfley, D. E., & Taylor, C. B. Healthy weight regulation and eating disorder prevention in high school students: a universal and targeted Web-based intervention. *Journal of Medical Internet Research* 2014, 16(2), e57. |
| 1. Liu, Z., Gao, P., Gao, A. Y., Lin, Y., Feng, X. X., Zhang, F., ... & Wu, Y. F. Effectiveness of a multifaceted intervention for prevention of obesity in primary school children in China: a cluster randomized clinical trial. *JAMA pediatrics* 2022, 176(1), e214375-e214375. |
| 1. Lubans, D. R., Morgan, P. J., Callister, R., & Collins, C. E. Effects of integrating pedometers, parental materials, and E-mail support within an extracurricular school sport intervention. *Journal of Adolescent Health* 2009, 44(2), 176-183. |
| 1. Lubans, D. R., Smith, J. J., Eather, N., Leahy, A. A., Morgan, P. J., Lonsdale, C., ... & Hillman, C. H. Time-efficient intervention to improve older adolescents’ cardiorespiratory fitness: findings from the ‘Burn 2 Learn’cluster randomised controlled trial. *British Journal of Sports Medicine* 2021, 55(13), 751-758. |
| 1. Neufeld, N. D. Outcome analysis of the BESTRONG childhood obesity treatment program: Effectiveness of an eight-week family-based childhood obesity program using an internet-based health tracker. *Childhood Obesity* 2016, 12(4), 227-236. |
| 1. Newton RL Jr, Marker AM, Allen HR, Machtmes R, Han H, Johnson WD, Schuna JM Jr, Broyles ST, Tudor-Locke C, Church TS. Parent-targeted mobile phone intervention to increase physical activity in sedentary children: randomized pilot trial. JMIR Mhealth Uhealth. 2014 Nov 10;2(4):e48. doi: 10.2196/mhealth.3420. |
| 1. Prochaska, J. J., & Sallis, J. F. A randomized controlled trial of single versus multiple health behavior change: promoting physical activity and nutrition among adolescents. *Health Psychology* 2004, 23(3), 314. |
| 1. Robbins, L. B., Ling, J., Sharma, D. B., Dalimonte-Merckling, D. M., Voskuil, V. R., Resnicow, K., ... & Pfeiffer, K. A. Intervention effects of “Girls on the Move” on increasing physical activity: A group randomized trial. *Annals of Behavioral Medicine* 2019, 53(5), 493-500. |
| 1. Robertson, W., Fleming, J., Kamal, A., Hamborg, T., Khan, K. A., Griffiths, F., ... & Thorogood, M. Randomised controlled trial evaluating the effectiveness and cost-effectiveness of' Families for Health', a family-based childhood obesity treatment intervention delivered in a community setting for ages 6 to 11 years. *Health Technology Assessment* 2017, 21(1), 1. |
| 1. Schoenfelder, E., Moreno, M., Wilner, M., Whitlock, K. B., & Mendoza, J. A. a mobile health intervention to increase physical activity for adolescents with ADHD. *Preventive medicine reports* 2017, 6, 210-213. |
| 1. Sousa, P., Martinho, R., Reis, C. I., Dias, S. S., Gaspar, P. J., Dixe, M. D. A., ... & Ferreira, R. Controlled trial of an mHealth intervention to promote healthy behaviours in adolescence (TeenPower): Effectiveness analysis. *Journal of Advanced Nursing* 2020, 76(4), 1057-1068. |
| 1. Straker, L. M., Howie, E. K., Smith, K. L., Fenner, A. A., Kerr, D. A., Olds, T. S., ... & Smith, A. J. The impact of Curtin University's activity, food and attitudes program on physical activity, sedentary time and fruit, vegetable and junk food consumption among overweight and obese adolescents: a waitlist controlled trial. *PLoS One* 2014, 9(11), e111954. |
| 1. Velicer, W. F., Redding, C. A., Paiva, A. L., Mauriello, L. M., Blissmer, B., Oatley, K., ... & Fernandez, A. C. Multiple behavior interventions to prevent substance abuse and increase energy balance behaviors in middle school students. *Translational behavioral medicine* 2013, 3(1), 82-93. |
| 1. Whittemore, R., Jeon, S., & Grey, M. An internet obesity prevention program for adolescents. *Journal of Adolescent Health* 2013, 52(4), 439-447. |
| **Not meeting outcome reporting inclusion criteria (n= 40)** |
| 1. Abraham, A. A., Chow, W. C., So, H. K., Yip, B. H. K., Li, A. M., Kumta, S. M., ... & Nelson, E. A. S. Lifestyle intervention using an internet-based curriculum with cell phone reminders for obese Chinese teens: a randomized controlled study. *PLoS One* 2015, 10(5), e0125673. |
| 1. Armstrong S, Mendelsohn A, Bennett G, Kemper AR. Texting Motivational Interviewing: A Randomized Controlled Trial of Motivational Interviewing Text Messages Designed to Augment Childhood Obesity Treatment. Child Obes 2018 Jan;14(1):4-10. |
| 1. Baños, R. M., Oliver, E., Navarro, J., Vara, M. D., Cebolla, A., Lurbe, E., ... & Botella, C. Efficacy of a cognitive and behavioral treatment for childhood obesity supported by the ETIOBE web platform. *Psychology, Health & Medicine* 2019, 24(6), 703-713. |
| 1. Barnes, C., Yoong, S. L., Nathan, N., Wolfenden, L., Wedesweiler, T., Kerr, J., ... & Grady, A. Feasibility of a Web-Based Implementation Intervention to Improve Child Dietary Intake in Early Childhood Education and Care: Pilot Randomized Controlled Trial. *Journal of Medical Internet Research* 2021, 23(12), e25902. |
| 1. Bech‐Larsen, T., & Grønhøj, A. Promoting healthy eating to children: a text message (SMS) feedback approach. *International Journal of Consumer Studies* 2013, 37(3), 250-256. |
| 1. Bianchi-Hayes, J., Schoenfeld, E., Cataldo, R., Hou, W., Messina, C., & Pati, S. Combining activity trackers with motivational interviewing and mutual support to increase physical activity in parent-adolescent dyads: longitudinal observational feasibility study*. JMIR pediatrics and parenting* 2018, 1(1), e8878. |
| 1. Blackman, K. C., Zoellner, J., Kadir, A., Dockery, B., Johnson, S. B., Almeida, F. A., ... & Estabrooks, P. A. Examining the feasibility of smartphone game applications for physical activity promotion in middle school students. *Games for health journal* 2015, 4(5), 409-419. |
| 1. Browne, S., Kechadi, M. T., O'Donnell, S., Dow, M., Tully, L., Doyle, G., & O'Malley, G. Mobile health apps in pediatric obesity treatment: process outcomes from a feasibility study of a multicomponent intervention. *JMIR mHealth and uHealth* 2020, 8(7), e16925. |
| 1. Cullen, K. W., Thompson, D., Boushey, C., Konzelmann, K., & Chen, T. A. Evaluation of a web-based program promoting healthy eating and physical activity for adolescents: teen choice: food and fitness. *Health education research* 2013, 28(4), 704-714. |
| 1. Cushing, C. C., Bejarano, C. M., Ortega, A., Sayre, N., Fedele, D. A., & Smyth, J. M. Adaptive mHealth intervention for adolescent physical activity promotion. *Journal of pediatric psychology* 2021, 46(5), 536-546. |
| 1. De-Jongh González, O., Tugault-Lafleur, C. N., Buckler, E. J., Hamilton, J., Ho, J., Buchholz, A., ... & Mâsse, L. C. The aim2be mhealth intervention for children with overweight or obesity and their parents: person-centered analyses to uncover digital phenotypes. *Journal of Medical Internet Research* 2022, 24(6), e35285. |
| 1. Fassnacht, D. B., Ali, K., Silva, C., Gonçalves, S., & Machado, P. P. Use of text messaging services to promote health behaviors in children. *Journal of nutrition education and behavior* 2015, 47(1), 75-80. |
| 1. Frenn, M., Malin, S., Bansal, N., Delgado, M., Greer, Y., Havice, M., ... & Schweizer, H. Addressing health disparities in middle school students' nutrition and exercise. Journal of Community Health Nursing 2003, 20(1), 1-14. |
| 1. Guthrie N, Bradlyn A, Thompson SK, Yen S, Haritatos J, Dillon F, Cole SW. Development of an accelerometer-linked online intervention system to promote physical activity in adolescents. PLoS One. 2015 May 26;10(5):e0128639. doi: 10.1371/journal.pone.0128639. |
| 1. Haerens, L., De Bourdeaudhuij, I., Maes, L., Vereecken, C., Brug, J., & Deforche, B. The effects of a middle-school healthy eating intervention on adolescents' fat and fruit intake and soft drinks consumption. *Public health nutrition* 2007, 10(5), 443-449. |
| 1. Hung, S. H., Hwang, S. L., Su, M. J., Lue, S. H., Hsu, C. Y., Chen, H. L., & Chen, H. S. An evaluation of a weight-loss program incorporating E-learning for obese junior high school students. *Telemedicine and e-Health* 2008, 14(8), 783-792. |
| 1. Jones, M., Luce, K. H., Osborne, M. I., Taylor, K., Cunning, D., Doyle, A. C., ... & Taylor, C. B. Randomized, controlled trial of an internet-facilitated intervention for reducing binge eating and overweight in adolescents. *Pediatrics* 2008, 121(3), 453-462. |
| 1. Lana, A., Faya-Ornia, G., & López, M. L. Impact of a web-based intervention supplemented with text messages to improve cancer prevention behaviors among adolescents: Results from a randomized controlled trial. *Preventive Medicine* 2014, 59, 54-59. |
| 1. Lau, E. Y., Lau, P. W., Chung, P. K., Ransdell, L. B., & Archer, E. Evaluation of an Internet–short message service–based intervention for promoting physical activity in Hong Kong Chinese adolescent school children: a pilot study. *Cyberpsychology, Behavior, and Social Networking* 2012, 15(8), 425-434. |
| 1. Mameli, C., Brunetti, D., Colombo, V., Bedogni, G., Schneider, L., Penagini, F., & Zuccotti, G. V. Combined use of a wristband and a smartphone to reduce body weight in obese children: Randomized controlled trial. *Pediatric Obesity* 2018, 13(2), 81–87. |
| 1. Mauriello, L. M., Ciavatta, M. M. H., Paiva, A. L., Sherman, K. J., Castle, P. H., Johnson, J. L., & Prochaska, J. M. Results of a multi-media multiple behavior obesity prevention program for adolescents. *Preventive medicine* 2010, 51(6), 451-456. |
| 1. Muzaffar, H., Chapman-Novakofski, K., Castelli, D. M., & Scherer, J. A. The HOT (Healthy Outcome for Teens) project. Using a web-based medium to influence attitude, subjective norm, perceived behavioral control and intention for obesity and type 2 diabetes prevention. *Appetite* 2014, 72, 82-89. |
| 1. Nawi, A. M., & Jamaludin, F. I. C. Effect of internet-based intervention on obesity among adolescents in Kuala Lumpur: a school-based cluster randomised trial*. The Malaysian Journal of Medical Sciences: MJMS* 2015, 22(4), 47. |
| 1. Ng, M., Wenden, E., Lester, L., Westgarth, C., & Christian, H. A mobile health intervention to encourage physical activity in children: a randomised controlled trial. *BMC pediatrics* 2022, 22(1), 276. |
| 1. Nguyen, B., Shrewsbury, V. A., O'connor, J., Steinbeck, K. S., Hill, A. J., Shah, S., ... & Baur, L. A. Two-year outcomes of an adjunctive telephone coaching and electronic contact intervention for adolescent weight-loss maintenance: the Loozit randomized controlled trial. *International Journal of Obesity* 2013, 37(3), 468-472. |
| 1. Paineau, D. L., Beaufils, F., Boulier, A., Cassuto, D. A., Chwalow, J., Combris, P., ... & Bornet, F. Family dietary coaching to improve nutritional intakes and body weight control: a randomized controlled trial. *Archives of pediatrics & adolescent medicine* 2008, 162(1), 34-43. |
| 1. Salahshoornezhad, S., Sohrabi, Z., Mani, A., Abdelbasset, W. K., Mehrabi, M., Zare, M., ... & Nouri, M. Effect of a multi-disciplinary program on anthropometric and biochemical parameters in obese and overweight elementary school girls: A randomized clinical trial. *Nutrition, Metabolism and Cardiovascular Diseases* 2022, 32(8), 1982-1989. |
| 1. Schiel, R., Beltschikow, W., Radón, S., Kramer, G., Schmiedel, R., Berndt, R. D., & Stein, G. Long-term treatment of obese children and adolescents using a telemedicine support programme. *Journal of Telemedicine and Telecare* 2008, 14(1), 13-16. |
| 1. Sirriyeh, R., Lawton, R., & Ward, J. Physical activity and adolescents: an exploratory randomized controlled trial investigating the influence of affective and instrumental text messages. *British journal of health psychology* 2010, 15(4), 825-840. |
| 1. TaverasElsie, M., & KemperAlex, R. Texting motivational interviewing: a randomized controlled trial of motivational interviewing text messages designed to augment childhood obesity treatment. Childhood Obesity 2018. |
| 1. Toscos, T., Faber, A., Connelly, K., & Upoma, A. M. Encouraging Physical Activity in Teens. *Pervasive Health* 2008, 218-221. |
| 1. Thornton, L., Gardner, L. A., Osman, B., Green, O., Champion, K. E., Bryant, Z., ... & Health4Life Team. A multiple health behavior change, self-monitoring mobile app for adolescents: development and usability study of the Health4Life app. *JMIR formative research* 2021, 5(4), e25513. |
| 1. Tripicchio, G. L., Kay, M., Herring, S., Cos, T., Bresnahan, C., Gartner, D., ... & Bass, S. B. Development and preliminary feasibility of iByte4Health: a mobile health (mHealth) pediatric obesity prevention intervention to engage parents with low-income of children 2–9 years. *Nutrients* 2021, 13(12), 4240. |
| 1. Van Woudenberg, T. J., Bevelander, K. E., Burk, W. J., Smit, C. R., Buijs, L., & Buijzen, M. A randomized controlled trial testing a social network intervention to promote physical activity among adolescents. *BMC public health* (2018), 18(1), 1-11. |
| 1. Verswijveren SJJM, Abbott G, Lai SK, Salmon J, Timperio A, Brown H, Macfarlane S, Ridgers ND. Mediators of Effects on Physical Activity and Sedentary Time in an Activity Tracker and Behavior Change Intervention for Adolescents: Secondary Analysis of a Cluster Randomized Controlled Trial *JMIR Mhealth Uhealth* 2022;10(8):e35261 |
| 1. Vidmar, A. P., Pretlow, R., Borzutzky, C., Wee, C. P., Fox, D. S., Fink, C., & Mittelman, S. D. An addiction model‐based mobile health weight loss intervention in adolescents with obesity. *Pediatric obesity* 2019, 14(2), e12464. |
| 1. White, M. A., Martin, P. D., Newton, R. L., Walden, H. M., York‐Crowe, E. E., Gordon, S. T., ... & Williamson, D. A. Mediators of weight loss in a family‐based intervention presented over the internet. *Obesity research* 2004, 12(7), 1050-1059. |
| 1. Williamson, D. A., Davis Martin, P., White, M. A., Newton, R., Walden, H., York-Crowe, E., ... & Ryan, D. Efficacy of an internet-based behavioral weight loss program for overweight adolescent African-American girls. *Eating and Weight Disorders-Studies on Anorexia, Bulimia and Obesity* 2005, 10, 193-203. |
| 1. Williamson, D. A., Walden, H. M., White, M. A., York‐Crowe, E., Newton Jr, R. L., Alfonso, A., ... & Ryan, D. Two‐year internet‐based randomized controlled trial for weight loss in African‐American girls. *Obesity* 2006, 14(7), 1231-1243. |
| 1. Zach, S., Raviv, T., & Meckel, Y. Using information communication technologies (ICTs) for motivating female adolescents to exercise/run in their leisure time. *Computers in Human Behavior* 2016, 60, 593-601. |
| **Study protocol (n= 8)** |
| 1. Babic, M. J., Morgan, P. J., Plotnikoff, R. C., Lonsdale, C., Eather, N., Skinner, G., ... & Lubans, D. R. Rationale and study protocol for ‘Switch-off 4 Healthy Minds’(S4HM): A cluster randomized controlled trial to reduce recreational screen time in adolescents. *Contemporary Clinical Trials* 2015, 40, 150-158. |
| 1. Barlow, S. E., Yudkin, J., Nelson, V., & Allicock, M. A. Dynamo Kids!/¡ Niños Dinámicos! A Web Site for Pediatric Primary Care Providers to Offer Parents of Children 6− 12 Years Old With Overweight and Obesity: Web Site Development and Protocol for Pilot Study. *Journal of Pediatric Health Care* 2023, 37(1), 17-24. |
| 1. Bendtsen M, Bendtsen P, Henriksson H, Henriksson P, Müssener U, Thomas K, Löf M The Mobile Health Multiple Lifestyle Behavior Interventions Across the Lifespan (MoBILE) Research Program: Protocol for Development, Evaluation, and Implementation *JMIR Res Protoc* 2020;9(4):e14894 |
| 1. Bendtsen, M., Seiterö, A., Bendtsen, P., Henriksson, H., Henriksson, P., Thomas, K., ... & Müssener, U. mHealth intervention for multiple lifestyle behaviour change among high school students in Sweden (LIFE4YOUth): protocol for a randomised controlled trial. *BMC public health* 2021, 21(1), 1-12. |
| 1. Delisle, C., Sandin, S., Forsum, E., Henriksson, H., Trolle-Lagerros, Y., Larsson, C., ... & Löf, M. A web-and mobile phone-based intervention to prevent obesity in 4-year-olds (MINISTOP): a population-based randomized controlled trial. *BMC public health* 2015, 15, 1-8. |
| 1. Ek, A., Delisle Nyström, C., Chirita-Emandi, A., Tur, J. A., Nordin, K., Bouzas, C., ... & Nowicka, P. A randomized controlled trial for overweight and obesity in preschoolers: the More and Less Europe study-an intervention within the STOP project. *BMC Public Health* 2019, 19(1), 1-13. |
| 1. Henriksson, H., Alexandrou, C., Henriksson, P., Henström, M., Bendtsen, M., Thomas, K., ... & Löf, M. MINISTOP 2.0: a smartphone app integrated in primary child health care to promote healthy diet and physical activity behaviours and prevent obesity in preschool-aged children: protocol for a hybrid design effectiveness-implementation study. *BMC Public Health* 2020, 20, 1-11. |
| 1. Sousa, P., Duarte, E., Ferreira, R., Esperanca, A., Frontini, R., Santos‐Rocha, R., ... & Marques, N. An mH ealth intervention programme to promote healthy behaviours and prevent adolescent obesity (TeenPower): a study protocol. *Journal of Advanced Nursing* 2019, 75(3), 683-691. |
| **Systematic reviews (n= 13)** |
| 1. Baumann H, Fiedler J, Wunsch K, Woll A, Wollesen B. mHealth Interventions to Reduce Physical Inactivity and Sedentary Behavior in Children and Adolescents: Systematic Review and Meta-analysis of Randomized Controlled Trials *JMIR Mhealth Uhealth* 2022;10(5):e35920 |
| 1. Böhm B, Karwiese S, Böhm H, Oberhoffer R. Effects of Mobile Health Including Wearable Activity Trackers to Increase Physical Activity Outcomes Among Healthy Children and Adolescents: Systematic Review *JMIR Mhealth Uhealth* 2019;7(4):e8298 |
| 1. Butler S, Sculley D, Santos D, Fellas A, Gironès X, Singh-Grewal D, Coda A. Effectiveness of eHealth and mHealth Interventions Supporting Children and Young People Living With Juvenile Idiopathic Arthritis: Systematic Review and Meta-analysis *J Med Internet Res* 2022;24(2):e30457 |
| 1. Champion, K. E., Parmenter, B., McGowan, C., Spring, B., Wafford, Q. E., Gardner, L. A., ... & Mewton, L. Effectiveness of school-based eHealth interventions to prevent multiple lifestyle risk behaviours among adolescents: a systematic review and meta-analysis. The Lancet Digital Health 2019, 1(5), e206-e221. |
| 1. Katherine E. Darling and Amy F. Sato. Systematic Review and Meta-Analysis Examining the Effectiveness of Mobile Health Technologies in Using Self-Monitoring for Pediatric Weight Management*. Childhood Obesity*. Oct 2017.347-355. |
| 1. He Z, Wu H, Yu F, Fu J, Sun S, Huang T, Wang R, Chen D, Zhao G, Quan M. Effects of Smartphone-Based Interventions on Physical Activity in Children and Adolescents: Systematic Review and Meta-analysis. *JMIR Mhealth Uhealth* 2021;9(2):e22601 |
| 1. Langarizadeh, M., Sadeghi, M., As’habi, A., Rahmati, P., & Sheikhtaheri, A. Mobile apps for weight management in children and adolescents; An updated systematic review. *Patient Education and Counseling* 2021, 104(9), 2181-2188. |
| 1. Ludwig K, Arthur R, Sculthorpe N, Fountain H, Buchan DS. Text Messaging Interventions for Improvement in Physical Activity and Sedentary Behavior in Youth: Systematic Review. *JMIR Mhealth Uhealth* 2018;6(9):e10799 |
| 1. Ridgers ND, McNarry MA, Mackintosh KA. Feasibility and Effectiveness of Using Wearable Activity Trackers in Youth: A Systematic Review *JMIR Mhealth Uhealth* 2016;4(4):e129 |
| 1. Rose, T., Barker, M., Jacob, C. M., Morrison, L., Lawrence, W., Strömmer, S., ... & Baird, J. A systematic review of digital interventions for improving the diet and physical activity behaviors of adolescents. *Journal of Adolescent Health* 2017, 61(6), 669-677. |
| 1. Turner, T., Spruijt‐Metz, D., Wen, C. F., & Hingle, M. D. Prevention and treatment of pediatric obesity using mobile and wireless technologies: a systematic review. *Pediatric obesity* 2015, 10(6), 403-409. |
| 1. Shin, Y., Kim, S. K., & Lee, M. Mobile phone interventions to improve adolescents' physical health: A systematic review and meta‐analysis. *Public Health Nursing* 2019, 36(6), 787-799. |
| 1. Yau KW, Tang TS, Görges M, Pinkney S, Kim AD, Kalia A, Amed S. Effectiveness of Mobile Apps in Promoting Healthy Behavior Changes and Preventing Obesity in Children: Systematic Review *JMIR Pediatr Parent* 2022;5(1):e34967 |

**Table S3.** Detailed intervention characteristics of included studies

| **Reference** | **Intervention characteristics** | | | **Outcome characteristics** | |
| --- | --- | --- | --- | --- | --- |
|  | **Intervention** | **Control** | **Duration (weeks)** | **Outcome** | **Instrument** |
| **App intervention** | | | | | |
| **AIMFIT - Direito et al. 2015** | IG1: Zombies, Run: Immersive App  IG2: Get Running: Non-immersive app  Both to to improve fitness and ability to run 5 km | Usual behaviour | 8 | Counts/min  Light PA (min/day)  MVPA(min/day)  Sedentary time (min/day) | Accelerometer  Accelerometer |
| **Exergame intervention** | | | | | |
| **Staiano et al. 2017** | Exergames focused on dancing (1 h per session, three times a week) | No-treatment | 12 | Light PA, Moderate PA, Vigorous PA (min/day)  Sedentary time (min/day) | Accelerometer  Accelerometer |
| **GameSquad – Staiano et al. 2018** | Gaming console with exergames, a gameplay curriculum  (1 h per session, three times a week) and video chat sessions with a fitness coach  (telehealth coaching). | Usual behaviour (wait-list design) | 24 | MVPA(min/day) | Accelerometer |
| **Multi-component intervention** | | | | | |
| **Switch-off 4 Healthy Minds - Babic et al. 2016** | S4HM Intervention included an interactive  seminar, eHealth messaging, a behavioral contract and parental newsletters | Usual behaviour | 24 | MVPA (min/day)  Screen-time (min/day) | Accelerometer  ASAQ Questionnaire (Adolescent Sedentary Activity Questionnaire) |
| **NEAT –**  **Dewar et al. 2013**  **Dewar et al. 2014**  **Lubans et al. 2012** | NEAT Girls intervention: enhanced school sport sessions, lunchtime  physical activity sessions, nutrition workshops, interactive educational  seminars, pedometers for self-monitoring, student handbooks,  parent newsletters, and text messages to reinforce and  encourage targeted health behaviors. | Usual behaviour (wait-list design) | 48 | Counts/min  MVPA (%)  Screen-time (min/day)  Sedentary time (min/day) | Accelerometer  Adolescent Sedentary Activity  Questionnaire |
| **Fit4life –**  **Huang et al. 2014** | Fit4Life web-and-text and phone counseling-based weight management interventions | Printed weight management materials once a month | 16 | MVPA (min/day) | Accelerometer |
| **ATLAS –**  **Lubans et al. 2016**  **Smith et al. 2014** | ATLAS (Active Teen Leaders Avoiding Screen-time) Intervention: professional development, fitness equipment for  schools, teacher-delivered physical activity sessions, lunch-time activity sessions, researcher-led seminars, a smartphone  application, and parental strategies. | Usual behaviour (wait-list design) | 20 | Counts/min  Screen-time (min/day) | Accelerometer  Adolescent Sedentary Activity  Questionnaire |
| **Mendoza et al. 2017** | Wearable PA-tracking device  and a peer-based virtual support group | Usual care | 10 | MVPA (min/day)  Sedentary time (min/day) | Accelerometer  Accelerometer |
| **PACEi-DP -Patrick et al. 2013** | Pace-Internet for Diabetes Prevention Intervention (PACEi-DP).  Website only (W); website, monthly group  sessions, and follow-up calls (WG); and website and SMS (WSMS). | Printed materials and three initial group sessions. | 48 | MVPA (min/week)  Sedentary time (hours/day) | 7-day physical activity recall interview, developed for the Stanford Five-City  Project  eight-item survey based on  a survey developed by Robinson |
| **Ruotsalainen et al. 2015** | Facebook-deliverded lifestyle counselling + PA self monitoring | Usual care | 12 | Light PA (min/day)  MVPA (min/day)  Sedentary time (min/day) | Accelerometer |
| **Thompson et al. 2016** | Pedometer + goal prompts + SDT-informed  texts (texts promoting satisfaction of the basic psychological  needs). | Usual behaviour (wait-list design) | 12 | Steps/day  MVPA (min/day) | Accelerometer |
| **Telephone delivered interventions** | | | | | |
| **HEAT –**  **Wright et al. 2013** | Healthy Eating and Activity Today (HEAT) study. Telephone counseling  intervention delivered by an automated interactive voice response system. The intervention  also included an electronic health record behavioral counseling tool | Usual behaviour (wait-list design) | 12 | Screen-time (hours/day) | Survey developed by Robinson |
| **Text messages intervention** | | | | | |
| **Newton et al. 2009** | Pedometer and regular motivational  text messages | Usual care | 12 | Steps/day  MVPA (min/week) | Pedometer  Self-reported physical activity over 7  days measured by a validated questionnaire |
| **Shapiro et al. 2008** | Weekly educational group sessions and monitoring, goal setting and personalized feedback through SMS. | Three educational group sessions. | 8 | Steps/day  Screen-time (min/day) | Pedometer  Pedometer and self-reported |
| **Web-based intervention** | | | | | |
| **Rheumates@Work -Armbrust et al. 2017**  **Lelieveld et al. 2010** | Rheumates@Work combination of internet-based  and individual instruction, supplemented with 4 group  sessions | Usual care | 17 | Light PA (min/day)  MVPA (min/day) | Accelerometer |
| **HELENA Study –**  **De Bourdeaudhuij et al. 2010**  **Cook et al. 2014** | Activ-O-Meter an internet-based computer-tailored  physical activity intervention | Generic advice | 12 | MVPA (min/week) | International Physical Activity  Questionnaire (IPAQ-A) |
| **FATaintPHAT -Ezendam et al. 2012** | FATaintPHAT. Web-based computer-tailored  intervention aiming to increase physical activity, decrease  sedentary behavior, and promote healthy eating | No-intervention | 96 | Steps/day  Screen-time (min/day) | Pedometer  Flemish validated questionnaire |
| **Families Reporting Every Step to**  **Health –**  **Guagliano et al. 2020** | Families Reporting Every Step to  Health. Web-based intervention, pedometers and weekly meetings aimed at increasing PA for  the whole family | No-intervention | 52 | MVPA (min/day)  Sedentary time (min/day) | Accelerometer  Accelerometer |
| **Haerens et al. 2006**  **Haerens et al. 2007** | IG2: Computer tailored  intervention for physical activity and tailored feedback plus PA promotion in schools  IG1: IG2 plus parent involvement | No-intervention | 96 | Light PA (min/day)  MVPA (min/day)  Screen-time (min/day) | Accelerometer  Accelerometer |
| **Slootmaker et al. 2010** | PAM COACH website with tailored PA advice and goal setting and accelerometer | Written information  brochure with brief general PA recommendations | 12 | Light PA (min/week)  MVPA (min/week)  Sedentary time (min/week) | Accelerometer  Accelerometer |

**Table S4.** GRADE Summary of findings

| **Certainty assessment** | | | | | | | **№ of patients** | | **Effect** | **Certainty** | **Importance** |
| --- | --- | --- | --- | --- | --- | --- | --- | --- | --- | --- | --- |
| **№ of studies** | **Study design** | **Risk of bias** | **Inconsistency** | **Indirectness** | **Imprecision** | **Other considerations** | **eHealth interventions** | **control** | **Absolute (95% CI)** |  |  |
| **Counts per minute (follow-up: range 2 months to 24 months; assessed with: Accelerometer)** | | | | | | | | | | | |
| 3 | randomised trials | very serious^a^ | not serious | not serious | serious^b^ | none | 192 | 210 | MD **16.11 lower** (122.76 lower to 90.53 higher) | ⨁◯◯◯ Very low | IMPORTANT |
| **Steps per day (follow-up: range 3 months to 24 months; assessed with: Pedometer)** | | | | | | | | | | | |
| 2 | randomised trials | serious^c^ | not serious | not serious | serious^b^ | none | 75 | 77 | MD **593.46 higher** (2102.27 lower to 3289.19 higher) | ⨁⨁◯◯ Low | CRITICAL |
| **MVPA (follow-up: range 2 months to 24 months; assessed with: Accelerometer)** | | | | | | | | | | | |
| 14 | randomised trials | serious^a^ | serious^d^ | not serious | not serious | none | 599 | 534 | MD **1.99 lower** (8.95 lower to 4.96 higher) | ⨁⨁◯◯ Low | CRITICAL |
| **LPA (follow-up: range 2 months to 24 months; assessed with: Accelerometer)** | | | | | | | | | | | |
| 5 | randomised trials | very serious^a^ | serious^d^ | not serious | serious^b^ | none | 209 | 148 | MD **3.98** **higher** (15.48 lower to 22.04 higher) | ⨁◯◯◯ Very low | CRITICAL |
| **Screen time (follow-up: range 2 months to 24 months; assessed with: Self-reported measurements)** | | | | | | | | | | | |
| 5 | randomised trials | very serious^a^ | not serious | not serious | not serious | none | 439 | 465 | MD **31.48 lower** (68.62 lower to 5.65 higher) | ⨁⨁◯◯ Low | CRITICAL |
| **Sedentary time (follow-up: range 2 months to 24 months; assessed with: Accelerometer)** | | | | | | | | | | | |
| 8 | randomised trials | very serious^a^ | serious^d^ | not serious | not serious | none | 443 | 358 | MD **33.12 lower** (57.27 lower to 8.97 lower) | ⨁◯◯◯ Very low | CRITICAL |

**CI:** confidence interval; **MD:** mean difference

a. Most of the included studies showed high risk of bias when assessed by Rob2 tool

b. Imprecision of the results may have been serious enough to downgrade the quality of evidence

c. Most of the included studies showed some concerns when assessed by RoB2 tool

d. Heterogeneity value I^2^ 50-75%

**Table S5.** Meta-regression

|  | Age | | | Percentage of women | | | |
| --- | --- | --- | --- | --- | --- | --- | --- |
|  | **n** | **ß (95%CI)** | **p** | **n** | **ß (95%CI)** | **p** |  |
| PA parameters | | | | | | | |
| Counts per minute | 3 | 18.61 (-288.58, 325.79) | 0.582 | 3 | -0.67 (-7.68, 6.34) | 0.437 |  |
| Steps per day | 2 | - | - | 2 | - | - |  |
| MVPA | 14 | 0.76 (-2.63, 4.15) | 0.634 | 14 | -0.25 (-0.68, 0.18) | 0.231 |  |
| Light PA | 5 | -1.85 (-12.69, 8.99) | 0.626 | 5 | -0.98 (-2.43, 0.48) | 0.123 |  |
| Sedentary behaviours parameters | | | | | | | |
| Screen-time | 5 | 2.60 (-26.21, 31.42) | 0.792 | 5 | 0.17 (-1.18, 1.52) | 0.717 |  |
| Sedentary time | 8 | 0.57 (-12.36, 13.50) | 0.918 | 8 | -0.78 (-1.79, 0.23) | 0.109 |  |

**Table S6.** Subgroup analysis by type of intervention

| **Outcome** | **Intervention type** | **Nº of trials** | **MD (95%CI)** | **I^2^ (%)** |
| --- | --- | --- | --- | --- |
| **Counts per minute** | **App intervention** | k=1 | 30.68 counts (-43.62 to 104.98) | - |
|  | **Multicomponent intervention** | k=2 | -32.39 counts (-362.35 to 297.57) | 54.8 |
|  | *Test for subgroup differences* | | *Q=1.88* | *p=0.170* |
| **Steps per day** | **Multicomponent intervention** | k=1 | 818 steps (-317.45 to 1953.45) | - |
|  | **Text messages intervention** | k=1 | 393 steps (-677.66 to 1463.66) | - |
|  | *Test for subgroup differences* | | *Q=0.28* | *p=0.594* |
| **MVPA** | **App intervention** | k=1 | 4.13 min/day (-6.51 to 14.77) | - |
|  | **Exergame intervention** | k=1 | **11.40 min/day (2.25 to 20.55)** | - |
|  | **Multicomponent intervention** | k=6 | -3.22 min/day (-6.76 to 0.31) | 0.0 |
|  | **Text messages intervention** | k=1 | 1.41 min/day (-25.91 to 28.73) | - |
|  | **Web-based intervention** | k=5 | -8.73 min/day (-32.63 to 15.17) | 93.7 |
|  | *Test for subgroup differences* | | *Q=10.96* | *p=0.027* |
| **LPA** | **App intervention** | k=1 | 0.48 min/day (-36.47 to 37.43) | **-** |
|  | **Multicomponent intervention** | k=1 | -1.00 min/day (-45.65 to 43.65) | - |
|  | **Web-based intervention** | k=3 | 2.62 min/day (-44.74 to 49.99) | 83.3 |
|  | *Test for subgroup differences* | | *Q=0.03* | *p=0.988* |
| **Screen time** | **Multicomponent intervention** | k=3 | **-26.40 min/day (-31.31 to -21.49)** | 0.0 |
|  | **Telephone delivered intervention** | k=1 | **-126.00 min/day (-240.50 to -11.50)** | **-** |
|  | **Text messages intervention** | k=1 | -8.10 min/day (-93.69 to 77.49) | **-** |
|  | *Test for subgroup differences* | | *Q=3.08* | *p=0.214* |
| **Sedentary time** | **App intervention** | k=1 | -6.09 min/day (-63.69 to 51.51) | - |
|  | **Multicomponent intervention** | k=4 | **-39.97 min/day (-78.55 to -1.40)** | 49.8 |
|  | **Web-based intervention** | k=3 | -35.15 min/day (-130.37 to 60.07) | 81.4 |
|  | *Test for subgroup differences* | | Q= 1.14 | p=0.567 |

**Table S7.** Egger’s test

|  | p |
| --- | --- |
| PA parameters |  |
| Counts per minute | 0.106 |
| Steps per day | - |
| MVPA | 0.484 |
| Light PA | 0.632 |
| Sedentary behaviour parameters |  |
| Screen-time | 0.504 |
| Sedentary time | 0.352 |

|  | Randomization process | Deviations from the intended interventions |  | Missing outcome data | Measurement of the outcome | Selection of the reported result | Overall bias |  |  |
| --- | --- | --- | --- | --- | --- | --- | --- | --- | --- |
| AIMFIT - Direito et al. 2015 |  |  |  |  |  |  |  |  | Low risk |
| Families Reporting Every Step to Health - Guagliano et al. 2020 |  |  |  |  |  |  |  |  | Some concerns |
| Fit4Life - Huang et al. 2014 |  |  |  |  |  |  |  |  | High risk |
| Staiano et al. 2017 |  |  |  |  |  |  |  |  |  |
| GameSquad -Staiano et al. 2018 |  |  |  |  |  |  |  |  |  |
| HEAT - Wright et al. 2013 |  |  |  |  |  |  |  |  |  |
| Mendoza et al. 2017 |  |  |  |  |  |  |  |  |  |
| Newton et al. 2009 |  |  |  |  |  |  |  |  |  |
| PACEi-DP - Patrick et al. 2013 |  |  |  |  |  |  |  |  |  |
| Rheumates@Work - Armbrust et al. 2017 |  |  |  |  |  |  |  |  |  |
| Ruotslalain et al. 2015 |  |  |  |  |  |  |  |  |  |
| Shapiro et al. 2008 |  |  |  |  |  |  |  |  |  |
| Slootmaker et al. 2010 |  |  |  |  |  |  |  |  |  |
| Thompson et al. 2016 |  |  |  |  |  |  |  |  |  |

**Figure S1.** Quality assessment of the included RCTs

|  | Randomization process | Recruitment | Deviations from intended interventions | Missing outcome data | Measurement of the outcome | Selection of the reported result | Overall risk of bias |  |  | |  | |
| --- | --- | --- | --- | --- | --- | --- | --- | --- | --- | --- | --- | --- |
| Activ-O-Meter - Cook et al. 2014 |  |  |  |  |  |  |  |  |  | Low risk | |  |
| ATLAS - Lubans et al. 2016 |  |  |  |  |  |  |  |  |  | Some concerns | |  |
| FATaintPHAT - Ezendam et al. 2012 |  |  |  |  |  |  |  |  |  | High risk | |  |
| Haerens et al. 2007 |  |  |  |  |  |  |  |  |  |  | |  |
| NEAT - Dewar et al. 2014 |  |  |  |  |  |  |  |  |  | |  | |
| Switch-off 4 Healthy Minds - Babic et al. 2016 |  |  |  |  |  |  |  |  |  | |  | |

**Figure S2.** Quality assessment of the included cluster RCTs

**
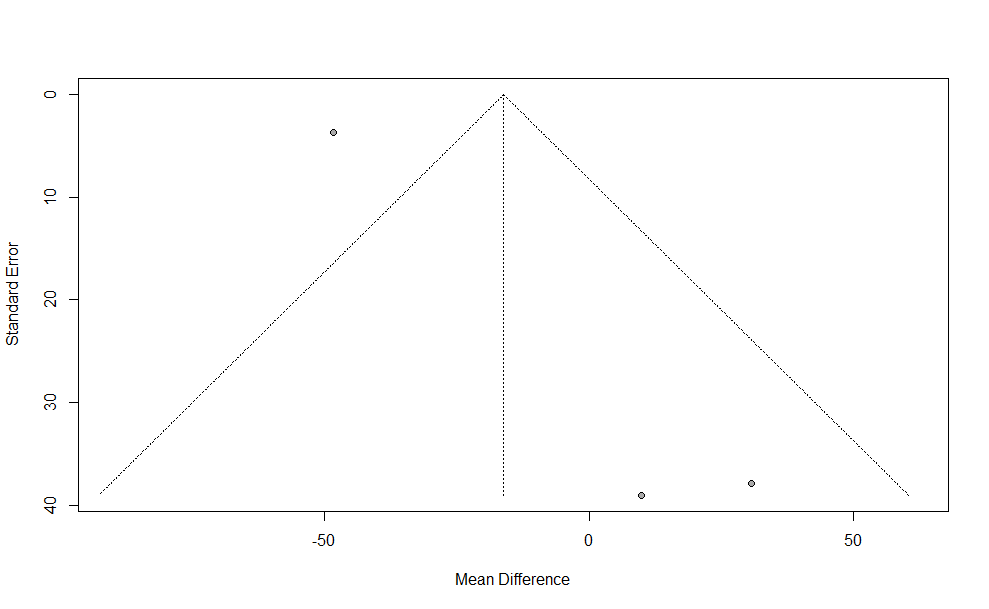
**

**Figure S3.** Funnel plot for counts per minute.

**
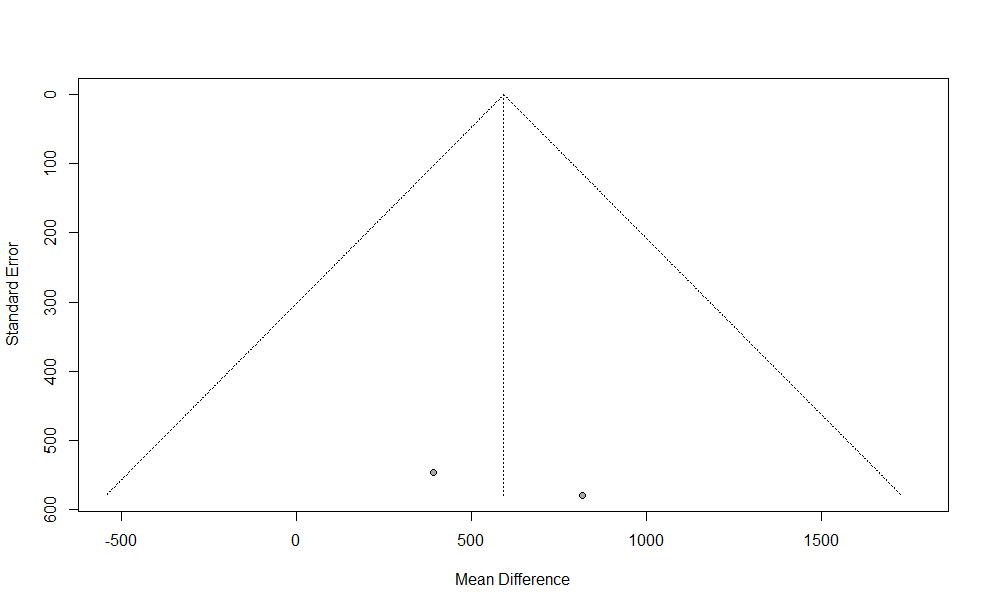
**

**Figure S4.** Funnel plot for steps per day.


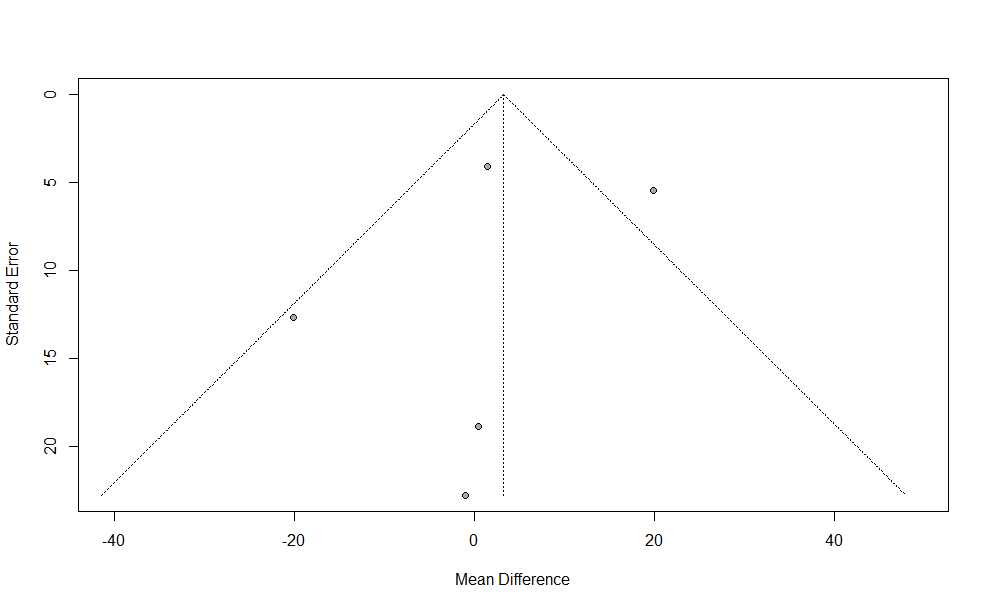


**Figure S5.** Funnel plot for light PA.

**
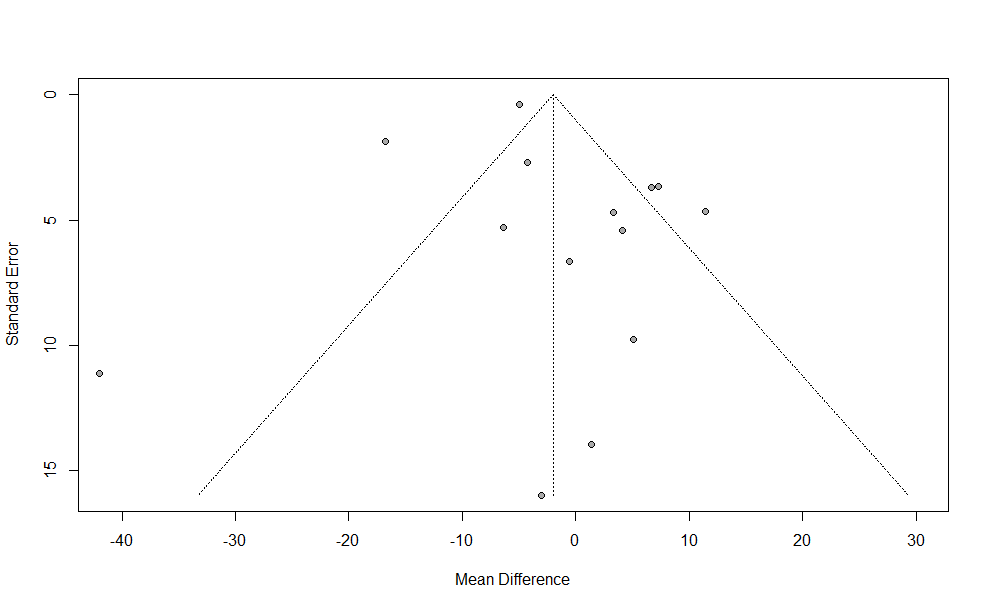
**

**Figure S6.** Funnel plot for MVPA.


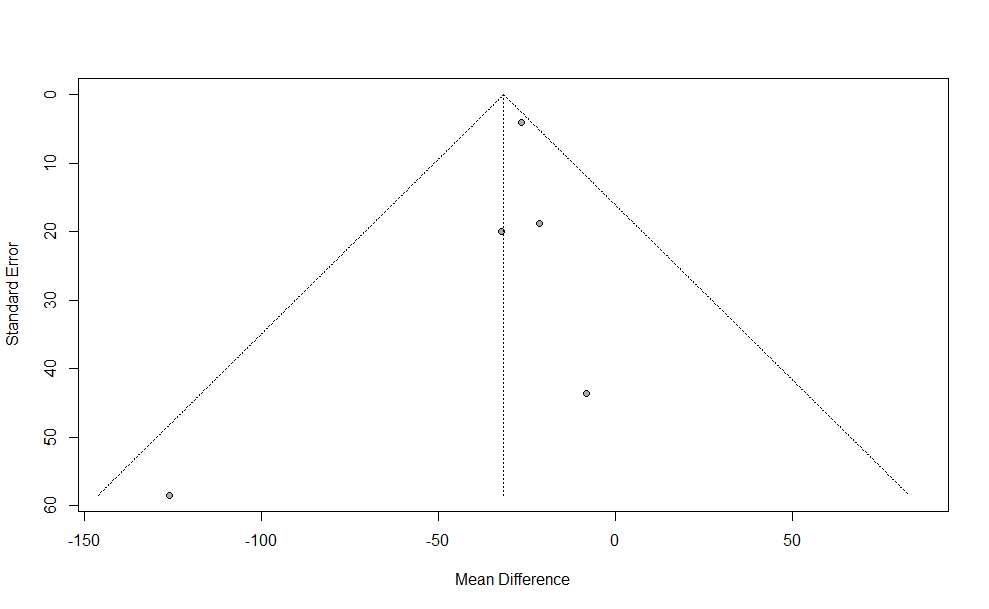


**Figure S7.** Funnel plot for screen-time.

**
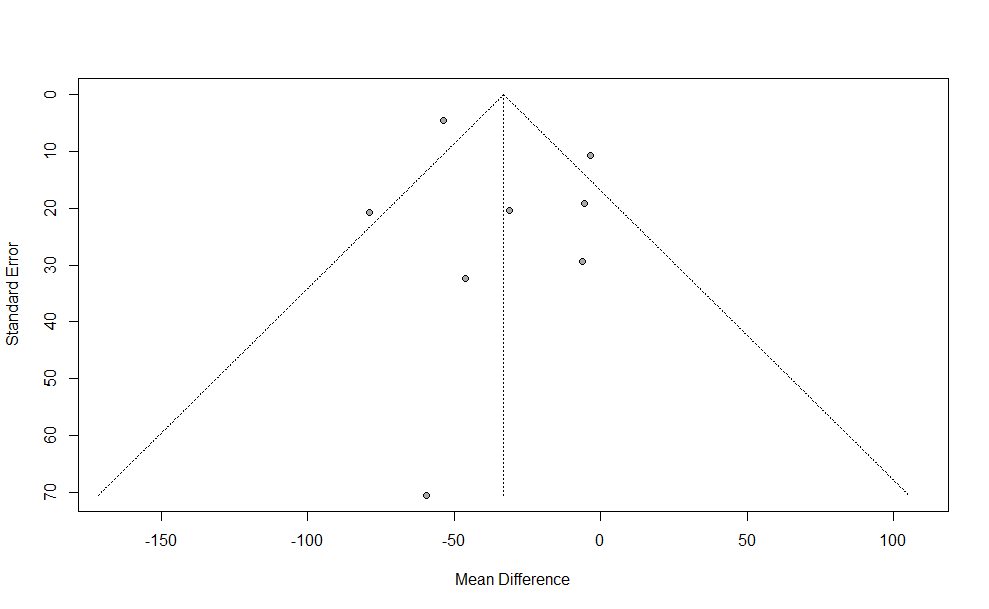
**

**Figure S8.** Funnel plot for sedentary time.
